# HTRA1-driven detachment of type I collagen from endoplasmic reticulum contributes to myocardial fibrosis in dilated cardiomyopathy

**DOI:** 10.1186/s12967-024-05098-7

**Published:** 2024-03-22

**Authors:** Hongjie Shi, Ming Yuan, Jie Cai, Lan Lan, Yumou Wang, Wei Wang, Jianliang Zhou, Bin Wang, Wenjun Yu, Zhe Dong, Dawei Deng, Qiaofeng Qian, Yang Li, Xianwu Zhou, Jinping Liu

**Affiliations:** 1 Department of Cardiovascular Surgery, Zhongnan Hospital of Wuhan University, 169 Donghu Road, Wuhan, 430071 People’s Republic of China; 2 Hubei Provincial Engineering Research Center of Minimally Invasive Cardiovascular Surgery, Wuhan, 430071 China; 3 Wuhan Clinical Research Center for Minimally Invasive Treatment of Structural Heart Disease, Wuhan, 430071 China; 4 Department of Radiology, Zhongnan Hospital of Wuhan University, Wuhan, 430071 China; 5 Department of Cardiovascular Ultrasound, Zhongnan Hospital of Wuhan University, Wuhan University, Wuhan, China

**Keywords:** Dilated cardiomyopathy, HTRA1, Myocardial fibrosis, ER stress, ER autophagy, COP II vesicle, Endoplasmic reticulum exit site (ERES)

## Abstract

**Background:**

The aberrant secretion and excessive deposition of type I collagen (Col1) are important factors in the pathogenesis of myocardial fibrosis in dilated cardiomyopathy (DCM). However, the precise molecular mechanisms underlying the synthesis and secretion of Col1 remain unclear.

**Methods and results:**

RNA-sequencing analysis revealed an increased HtrA serine peptidase 1 (HTRA1) expression in patients with DCM, which is strongly correlated with myocardial fibrosis. Consistent findings were observed in both human and mouse tissues by immunoblotting, quantitative reverse transcription polymerase chain reaction (qRT-PCR), immunohistochemistry, and immunofluorescence analyses. Pearson’s analysis showed a markedly positive correlation between HTRA1 level and myocardial fibrosis indicators, including extracellular volume fraction (ECV), native T1, and late gadolinium enhancement (LGE), in patients with DCM. In vitro experiments showed that the suppression of HTRA1 inhibited the conversion of cardiac fibroblasts into myofibroblasts and decreased Col1 secretion. Further investigations identified the role of HTRA1 in promoting the formation of endoplasmic reticulum (ER) exit sites, which facilitated the transportation of Col1 from the ER to the Golgi apparatus, thereby increasing its secretion. Conversely, HTRA1 knockdown impeded the retention of Col1 in the ER, triggering ER stress and subsequent induction of ER autophagy to degrade misfolded Col1 and maintain ER homeostasis. In vivo experiments using adeno-associated virus-serotype 9-shHTRA1-green fluorescent protein (AAV9-shHTRA1-GFP) showed that HTRA1 knockdown effectively suppressed myocardial fibrosis and improved left ventricular function in mice with DCM.

**Conclusions:**

The findings of this study provide valuable insights regarding the treatment of DCM-associated myocardial fibrosis and highlight the therapeutic potential of targeting HTRA1-mediated collagen secretion.

**Supplementary Information:**

The online version contains supplementary material available at 10.1186/s12967-024-05098-7.

## Introduction

Dilated cardiomyopathy (DCM) is a progressive and debilitating cardiovascular disorder characterized by the enlargement of ventricular chambers and impaired systolic function. DCM is a leading cause of heart failure and results in notable morbidity and mortality worldwide [1]. Myocardial fibrosis is a prominent pathological feature among the multifactorial and complex causes of DCM [2]. When the myocardium undergoes damage or chronic inflammation, cardiac cells, particularly myocardial interstitial cells, such as cardiac fibroblasts, generate excess collagen [3, 4]. Excessive collagen deposition leads to myocardial tissue stiffness and loss of elasticity, thereby affecting normal contraction and relaxation functions of the heart [5].

Collagen serves as the primary extracellular component of connective tissues and is the most prevalent protein found in animals, accounting for approximately 30% of the total protein content [6, 7]. The extracellular matrix (ECM) of the myocardium primarily consists of type I collagen (Col1) and type III collagen (Col3), which structurally support the myocardium and play vital roles in myocardial function [8]. Many studies have indicated that during the progression of myocardial fibrosis, both the transcriptional and translational levels of Col1 are considerably higher than those of other collagen types [9]. The synthesis of Col1 initiates with the production of procollagen polypeptide chains comprising N- and C-terminal propeptides. Subsequently, these procollagen chains are transported to the endoplasmic reticulum (ER), where they undergo molecular chaperon-assisted trimeric assembly [10, 11]. The assembled and properly folded procollagen is then transported to the Golgi apparatus via specific transport molecules. Subsequently, mature procollagen is secreted into the extracellular space, where the N- and C-terminal propeptides are enzymatically cleaved, leading to the formation of mature collagen [12, 13]. However, misfolded or improperly transported collagen within the ER is eliminated through autophagy–lysosomal mechanisms to maintain ER homeostasis [14–16]. The present research has considerably advanced our understanding of the modification, extracellular cleavage, and crosslinking mechanisms of Col1. However, information regarding the precise process of Col1 transportation from the ER to the Golgi apparatus is limited.

The coat protein complex II (COPII) vesicles facilitate the anterograde transport of secretion-ready cargo, such as collagen, from the ER to the Golgi apparatus [17]. The COPII-coated vesicles are formed at the ER exit sites (ERES). When the secretory cargo accumulates at ERES, Sar1 (in its active form, Sar1–guanine triphosphate [GTP]) is inserted into the ER membrane to recruit the Sec23/24 dimer. Thus, a Sar1–GTP/Sec23/Sec24 trimeric complex is assembled on the cytoplasmic surface of the ER membrane, which forms the inner layer of the COPII-coated vesicles [18, 19]. The COPII transport pathway, particularly the ERES, must adapt to changes in the external environment, as the flux and types of protein secretion vary [20]. However, COPII vesicles, with an average diameter of 60–80 nm, are insufficient to accommodate the secretion of large proteins, such as procollagen (300–400 nm).

Recent studies have revealed that overexpression of the Transport and Golgi organization protein 1 (TANGO1), located at the ERES, can expand ERES and facilitate the transport of large proteins, including collagen, to the Golgi apparatus. According to Maeda, TANGO1 coordinates the regulation of ERES size by recruiting SEC16A to promote the transport of large cargo [21]. SEC16A is a large peripheral membrane protein with a molecular weight of 250 kDa that is expressed in nearly all eukaryotic cells. It plays a crucial role in ERES formation and its loss or mutation can disrupt ERES formation and hinder the assembly of COPII vesicles [22, 23]. Farhan et al. reported that silencing SEC16A inhibited the number or intensity of ERES and altered the secretion flux to adapt to changes in acute or chronic cargo loads [20]. Therefore, targeting ERES regulation and COPII vesicle formation may improve tissue fibrosis by affecting collagen secretion and transport. It is unclear how molecules involved in assisting collagen secretion, such as SEC16A and TANGO1, are recruited to the ER and initiate ERES adaptation in DCM. Several cargo receptors involved in the specific transport of secretory proteins have been identified. HRD1 promotes COPII vesicle formation by recruiting SEC23A, which increases Col1 secretion and contributes to the progression of kidney fibrosis [24]. The thyroid adenoma-associated gene facilitates the interaction between the programmed cell death ligand 1 (PDL1), acting as a cargo protein, and SEC24A, thereby driving the Golgi residency of PDL1 [25]. The characterization of specific cargo receptors that mediate collagen transport in DCM contributes to the precise regulation of collagen synthesis and secretion, which affects the progression of myocardial fibrosis.

In case the assembly of ERES and COPII vesicle fails, the cargo is blocked within the ER, leading to ER stress, which induces ER autophagy and ER-associated degradation (ERAD) to eliminate the cargo. ER stress refers to the molecular and biochemical changes that occur when the balance between the morphology and function of the ER is disrupted, which leads to impaired protein processing and transport. This disruption leads to the accumulation of unfolded or misfolded proteins in the ER. Cells possess mechanisms that alleviate ER stress and restore normal ER function; however, excessive ER stress can contribute to disease progression [26, 27]. Autophagy is a cellular response where the damaged proteins are transported to the lysosomes for degradation [28]. Selective autophagy focuses on the degradation of specific substrates and plays a crucial role in maintaining cellular homeostasis [29]. ER autophagy, also known as reticulophagy, is a form of selective autophagy that helps maintain ER stability [30, 31]. The present research has only partially revealed the relationship between ER stress and ER autophagy [32–34]; however, the molecular mechanisms underlying the autophagic lysosomal degradation of Col1, which was discovered by Ishida et al. in 2009 to be potentially activated by ER stress rather than ERAD [15], have not been well elucidated.

Herein, we discovered that the HtrA serine peptidase 1 (HTRA1) level was considerably increased in DCM, and it was strongly associated with myocardial fibrosis. HTRA1 is a member of the HtrA family of serine proteases and is extensively expressed in various tissues and organs of the human body. Recent studies suggest that HTRA1 functions as a serine protease that targets ECM proteins, including aggrecan [35], chondroadherin [36], and fibronectin [37]. However, these findings provide only a superficial understanding and do not reveal the detailed molecular mechanisms underlying their effects on ECM remodeling. Yamawaki et al. reported that HTRA1 was significantly upregulated in scar fibroblasts and promoted the progression of scar fibrosis by increasing fibroblast proliferation and remodeling the scar matrix [38]. Oxidative and ER stress can induce HTRA1 upregulation in various diseases, both of which directly contribute to myocardial fibrosis [39, 40].

We identified HTRA1 as a crucial molecule involved in the synthesis and secretion of Col1. HTRA1 recruits SEC16A to facilitate ERES formation, which selectively binds and transports Col1 to the Golgi apparatus and promotes its secretion. This process accelerates the progression of myocardial fibrosis in patients with DCM. Loss of HTRA1 leads to ER stress and subsequent autophagy, which aids in the degradation of residual procollagen within the ER while maintaining its relative homeostasis. By targeting the rate-limiting steps of Col1 secretion, we successfully ameliorated the myocardial fibrosis process in DCM. The findings of this study offer novel insights into the treatment of DCM-associated myocardial fibrosis.

## Materials and methods

### Data acquisition and preprocessing

Six human DCM and one doxorubicin (Dox) mice RNA-sequencing datasets used in this study were obtained from Gene Expression Omnibus (GEO; http://www.ncbi.nlm.nih.gov/geo) (Additional file 1: Table S1). All expression matrix were successfully downloaded and annotated. In cases where multiple probes were associated with the same gene symbol, the average value was utilized as the ultimate value, and all data were logarithmically normalized. Although six human DCM datasets were used to compare HTRA1 expression between non-failing and DCM tissues, only GSE116250 was selected for further analysis benefiting its greater sequencing depth (42,251 genes were identified). X-Cell [38] and Estimate R packages [41] were performed to calculate the tissue stromascores and MCPCounter R package [42] was used to estimate the fibroblasts abundance. In addition, Gene Set Enrichment Analysis (GSEA) software (Version: 4.3.2) [43] was launched to explore HTRA1 related pathways based on hallmark gene sets.

### Human heart samples

Twenty-four human DCM samples were derived from DCM patients who received heart transplantation at the Cardiovascular Surgery Department, Zhongnan Hospital of Wuhan University and all patients reported DCM as a result of postoperative pathological examination. Non-failing heart tissues were obtained from six donors. Additionally, all the clinical information of DCM patients such as myocardial fibrosis indicators (ECV, native T1 and LGE) and cardiac function parameters (Table 1) were also collected. All studies involving human heart tissue have obtained informed consent from patients and approval from the Ethics Committee of Zhongnan Hospital of Wuhan University (2022075K).

**Table 1:** Baseline information of DCM patients

Factor	Total no. of participants (n = 24)	Low HTRA1 expression (n = 12)	High HTRA1 expression (n = 12)	p-value
Gender				0.22
Male	21 (87.5%)	12 (100%)	9 (75%)	
Female	3 (12.5%)	0 (0%)	3 (25%)	
Age (year)	44.08 ± 14.38	42.75 ± 14.4	45.42 ± 14.87	0.66
BNP	10.01 ± 1.03	9.45 ± 0.81	10.65 ± 0.89	**0.01**
NT-proBNP	12.06 ± 1.67	11.83 ± 1.89	12.28 ± 1.47	0.71
HS-TnI	5.69 ± 3.27	5.65 ± 3.27	5.73 ± 3.44	0.96
Height (m)	1.67 ± 0.11	1.67 ± 0.14	1.67 ± 0.06	0.99
Weight (kg)	63 ± 19.9	65.75 ± 20.19	60 ± 20.1	0.5
BMI (kg/m^2^)	22.16 ± 5.45	22.92 ± 4.6	21.32 ± 6.38	0.49
LVEF (%)	25 ± 6.79	24.42 ± 6.95	25.58 ± 6.88	0.68
NYHA				0.48
III	2 (8.3%)	2	10	
IV	22 (92.7%)	0	12	
EDV(ml)	433 ± 136.7	420.2 ± 150	447.1 ± 126.4	0.65
ESV(ml)	366.9 ± 129.5	350.4 ± 139.6	384.9 ± 121.5	0.54
SV(ml)	66.01 ± 21.1	69.76 ± 26.18	61.93 ± 13.79	0.39
LVEDVi (ml/m^2^)	245.9 ± 63.75	228.9 ± 63.07	264.5 ± 61.96	0.19
LVESVi (ml/m^2^)	207.3 ± 61.1	189 ± 58.24	227.3 ± 60.36	0.14
LVEF (%) CMR	16.1 ± 6.42	17.59 ± 7.86	14.46 ± 4.14	0.25
CI l/(min m^2^)	2.96 ± 1.57	3.27 ± 2.09	2.62 ± 0.61	0.33
SV(ml/m^2^)	39.69 ± 16.33	42.06 ± 21.35	37.09 ± 8.45	0.48
LV mass(g)	176.1 ± 65.8	178.4 ± 59.68	173.7 ± 74.8	0.87
LVMi (g/m^2^)	99.58 ± 31	97.04 ± 21.65	102.4 ± 39.78	0.69
HCT (%)	40.64 ± 5.85	42.08 ± 6.24	39.2 ± 5.29	0.24
Native T1 (ms)	1355 ± 58.26	1327 ± 45.92	1383 ± 57.2	**0.015**
ECV (%)	35.69 ± 5.93	32.86 ± 5.21	38.52 ± 5.37	**0.016**
LGE				0.64
LGE(−)	6 (25%)	4 (33.3%)	2 (16.7%)	
LGE( +)	18 (75%)	8 (66.7%)	10 (83.3%)	
LGE percent (%)	11.45 ± 14.11	5.15 ± 7.67	17.74 ± 16.46	**0.025**

Bold value represents *p* < 0.05 and has statistical significance

### Animals

C57BL/6J mice (male, 6–9 weeks) were purchased from Vital River Laboratories (Beijing, China). To induce a DCM model, the mice were subjected to alternate-day intraperitoneal injections of Dox (solution in saline, dosed at 4 mg∙kg-1, administered at a volume of 0.1 ml/10 g body weight) for two consecutive weeks. After a three-week drug withdrawal period, ultrasound analysis was conducted. Only the mice whose left ventricular end-diastolic diameter (LVEDd) fell within the 95% confidence interval were selected for further analysis as DCM mice. To generate HTRA1 knockdown mice, AAV9-shHTRA1-GFP virus (1.5*10^11^/150 μl per mouse) was injected via the tail vein for three weeks prior to constructing the DCM mouse model. All animals were sacrificed at the conclusion of the experiment and heart tissues and blood samples were gathered. The experimental protocol involving animals was thoroughly reviewed and ethically approved by the Institutional Animal Care and Use Committee of Zhongnan Hospital, Wuhan University.

### Histological staining analysis

Fresh heart tissues from both humans and mice were fixed using a 4% paraformaldehyde solution and then embedded in paraffin. Following dehydration and embedding, the tissue wax blocks were horizontally sectioned to facilitate subsequent experiments. Masson's staining was employed to assess the extent of tissue collagen deposition, while immunohistochemistry experiments were conducted to evaluate the expression levels of HTRA1 and fibrotic markers. Tissue immunofluorescence detection was utilized to examine variations in HTRA1 expression and tissue localization. Fiji ImageJ software (v1.53) was performed for statistical analysis of the captured images.

### Quantitative real-time PCR (qRT-PCR)

RNA extraction from tissues and cardiac fibroblasts was carried out using TRIzol reagent (Vazyme, China). Subsequently, the RNA was reverse transcribed into cDNA using the HiScript® Q RT SuperMix kit (Vazyme, China) following the manufacturer's instructions. Real-time PCR was performed on an Applied Biosystems Instrument using the ChamQTM SYBR® qPCR Master Mix (Vazyme, China). The relative expression levels of the target genes were determined using the 2−ΔΔCt method. The primer sequences used in this study are provided in Additional file 1: Table S2.

### Immunoblots and immunoprecipitation

The heart tissue and cells were thoroughly lysed using RIPA lysis buffer containing 1% protease inhibitor. Subsequently, the lysates were centrifuged at 12,000 rpm for 10 min at 4 °C, and the resulting supernatant was collected. Protein quantification was carried out using the BCA assay kit following the manufacturer's instructions, and the appropriate 5 × loading buffer was added. Finally, the mixture was boiled in a 95 °C metal bath for 10 min to obtain the protein samples.

Immunoprecipitation assays were performed according to the previously described method [44]. Briefly, 10 μl of protein A/G agarose beads (AA104307; Bestchrom, Shanghai, China) were incubated with the protein lysate for 1 h to facilitate purification. Subsequently, the beads were incubated overnight at 4 °C with 20 μl of protein A/G agarose beads containing 1 μl of antibodies. The immunoprecipitates bound to the beads surface were eluted using 2 × SDS loading buffer and then subjected to boiling at 95 °C for 15 min. Western blot analysis was conducted to detect protein expression. Detailed information regarding the antibodies utilized in this study can be found in Additional file 1: Table S3.

### Determination of Col1 concentration

To obtain the cell supernatant, collect 500 μl of the cell culture medium after intervening with cardiac fibroblasts, and centrifuge it at 4 °C and 6000 rpm for 10 min to remove any cellular debris. The concentration of the Col1 protein in cell supernatant was determined using the I-type collagen α1 enzyme-linked immunosorbent assay kit, following the instructions provided by the manufacturer (E-EL-R0041c, Elabscience).

### AAV9-shHTRA1-GFP construction

Mouse shHTRA1 sequence were inserted into an AAV9-based plasmid, which was further utilized for packaging AAV9 viruses. Prior to the construction of DCM mice, mice were injected with AAV9 virus via the tail vein at a dose of 1.5*1011 TU/mice three week in advance.

### Isolation and cultivation of neonatal rat primary cardiac fibroblasts (mCFs)

First, newborn rats (1–2 day) were pre-purchased from the Hubei Provincial Center for Disease Control and Prevention. Prior to initiating cardiac dissection, the mice were thoroughly wiped with 75% alcohol to ensure proper disinfection. Within a sterile laminar flow hood, aseptic surgical instruments were used to swiftly dissect and isolate the heart, which was then thoroughly rinsed with PBS buffer to eliminate any remaining blood. Subsequently, the surgical tools were changed, the atria and residual blood vessels were excised, and the ventricles were uniformly divided into tissue fragments of approximately 1 mm in diameter. These isolated ventricular tissues were placed in a tube containing around 8 ml of 0.125% trypsin solution and incubated on a shaker at 37 ℃ for 8 min. The resulting supernatant was collected and transferred to another tube containing 10 ml of DMEM culture medium supplemented with 50% FBS to neutralize the trypsin. This digestion process was repeated 5–7 times until complete digestion of the cardiac tissue was achieved. The digested solution was then filtered through a 70 μm mesh filter, followed by centrifugation at 1500 rpm for 5 min, and the supernatant was discarded. To the cell pellet, 3 ml of red blood cell lysis buffer was added, thoroughly lysed for 3 min, and then an equal volume of DMEM culture medium was added to halt the lysis. The mixture was centrifuged at 1500 rpm for 5 min, and the supernatant was discarded. The cell pellet was resuspended in DMEM culture medium containing 10% FBS. The resuspended cell suspension was evenly spread onto a 10 cm culture dish, and after 2 h, the supernatant was discarded and replaced with fresh culture medium. The adherent cells were identified as cardiac fibroblasts. Considering the propensity of cardiac fibroblasts to differentiate, only cardiac fibroblasts from the second to third passage were utilized in this study.

### Echocardiography

Small Animal Ultrasound Imaging System (VINNOD86OLAB, VINNO, China) is used for performing echocardiography to assess the cardiac function of mice. To prevent cardiac functional alterations triggered by stress-induced hair removal, all animals underwent depilation of the chest region using depilatory cream a day before conducting echocardiography. Mice were subjected to gas anesthesia with isoflurane and securely immobilized on a detection board. Cardiac function parameters in mice, such as LVEDd, left ventricular end-systolic diameter (LVEDs), left ventricular ejection fraction (LVEF), and fractional shortening (FS), were measured using M-mode in the long-axis view of the left ventricle.

### Immunofluorescence staining

Six-well plates were prepared with coverslips, and each coverslip was seeded with 200,000 cardiac fibroblasts. The coverslips were carefully collected and subjected to three washes with PBS buffer. Next, the cells were fixed with 4% paraformaldehyde at room temperature for 15 min. After thorough rinsing with PBS buffer, permeabilization was carried out using a solution containing 0.5% Triton-100 at room temperature for 20 min. Subsequently, the coverslips were blocked with a solution containing 5% BSA at room temperature for 1 h. Following the blocking step, the coverslips were incubated overnight at 4 °C with the primary antibody, followed by incubation at room temperature for 1 h with the corresponding secondary antibody. Finally, the coverslips were mounted on glass slides using a mounting medium containing DAPI. Confocal fluorescence microscopy (Leica) was utilized for the visualization and capture of fluorescent images.

### siRNA transfection

Cardiac fibroblasts were transfected with negative control (NC) and HTRA1 or SEC16A small interfering RNA (siRNA) using Lipofectamine 3000, following the manufacturer's instructions. The sequences of siRNAs used in this study are as follows: si-HTRA1#1: sense: 5′-GGUUCACAUUGAACUUUAUTT-3′, antisense: 5′-AUAAAGUUCAAUGUGAACCTT-3′; si-HTRA1#2: sense: 5′-GCUGAAGAAUGGAGCGACUTT-3’, antisense: 5′-AGUCGCUCCAUUCUUCAGCTT-3′; si-HTRA1#3: sense: 5′-GGGCAUCUCCUUCGCAAUUTT-3′, antisense: 5′-AAUUGCGAAGGAGAUGCCCTT-3′; si-SEC16A: sense: 5′-GCCACAGUGUGAGAAUGUATT-3′, antisense: 5′-UACAUUCUCACACUGUGGCTT-3′.

### Plasmid transfection

Flag-HTRA1 was prepared by cloning the digested PCR product into the pCDNA3.1( +) vector. Due to the large molecular weights of Col1 (222KDa) and SEC16A (260KDa), we were unable to successfully construct their full-length plasmids. Cardiac fibroblasts were transfected with empty vector and Flag-HTRA1 using Lipofectamine 3000 and P3000, following the manufacturer's instructions.

### Statistical analyses

All results are presented as the mean ± SD. Statistical comparisons between two groups were conducted using either Student’s t-test (for data with normal distribution and homogeneity of variance) or the Mann–Whitney test (for data that were not normally distributed or lacked homogeneity of variance). Correlation analysis is performed through Pearson’s chi-squared test. Graphpad Prism software (version: 9.5) is performed to analyze data in this study.

## Results

### HTRA1 is up-regulated and associated with myocardial fibrosis in DCM

**Fig. 1:**
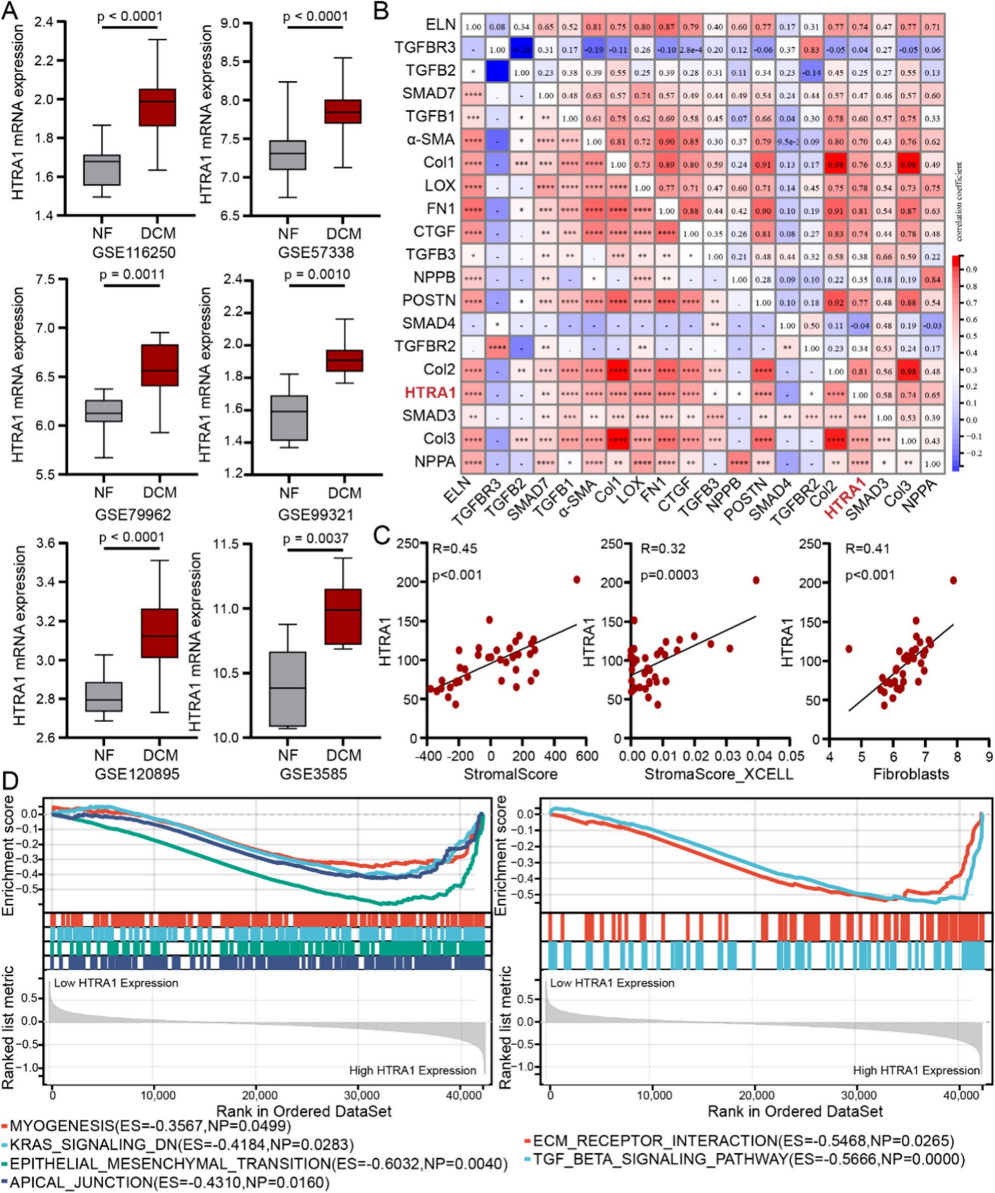
RNA sequencing analysis revealed the positive correlation between HTRA1 and myocardial fibrosis in DCM. **A** Differential analysis of HTRA1 expression between DCM and NF patients based on six different datasets (n: GSE116250: DCM:NF = 37:14; GSE57388: DCM:NF = 82:136; GSE79962: DCM:NF = 9:11; GSE99321: DCM:NF = 7:7; GSE120895: DCM:NF = 47:8; GSE3585: DCM:NF = 7:5). **B** The correlation analysis between HTRA1 and fibrogenic genes such as α-SMA, Col1, Fib and CTGF (*p < 0.05, **p < 0.01, ***p < 0.001, ****p < 0.0001). **C** Scatter plots showing the correlation between HTRA1 mRNA expression and tissue stromascores and fibroblasts abundance (Pearson’s chi-squared test was performed) **D** Hallmark and KEGG pathway enrichment analysis for HTRA1 using GSEA

To investigate the mRNA expression of HTRA1 in DCM, six datasets (Additional file 1: Table S1) obtained from GEO database were reanalyzed. RNA sequencing analysis revealed a significant up-regulation of HTRA1 in DCM (Fig. 1A). Furthermore, a Pearson correlation analysis demonstrated a significant positive correlation between HTRA1 expression and key molecules associated with myocardial fibrosis, such as Col1, α-SMA, and CTGF (Fig. 1B). Additionally, HTRA1 exhibited strong correlations with stromascore and fibroblast content (Fig. 1C). GSEA indicated that HTRA1 was significantly enriched in functions related to epithelial-mesenchymal transition, TGFβ signaling pathway, and ECM receptor interaction (Fig. 1D). These findings strongly suggest that HTRA1 plays a crucial role in the process of myocardial fibrosis in individuals with DCM.

**Fig. 2:**
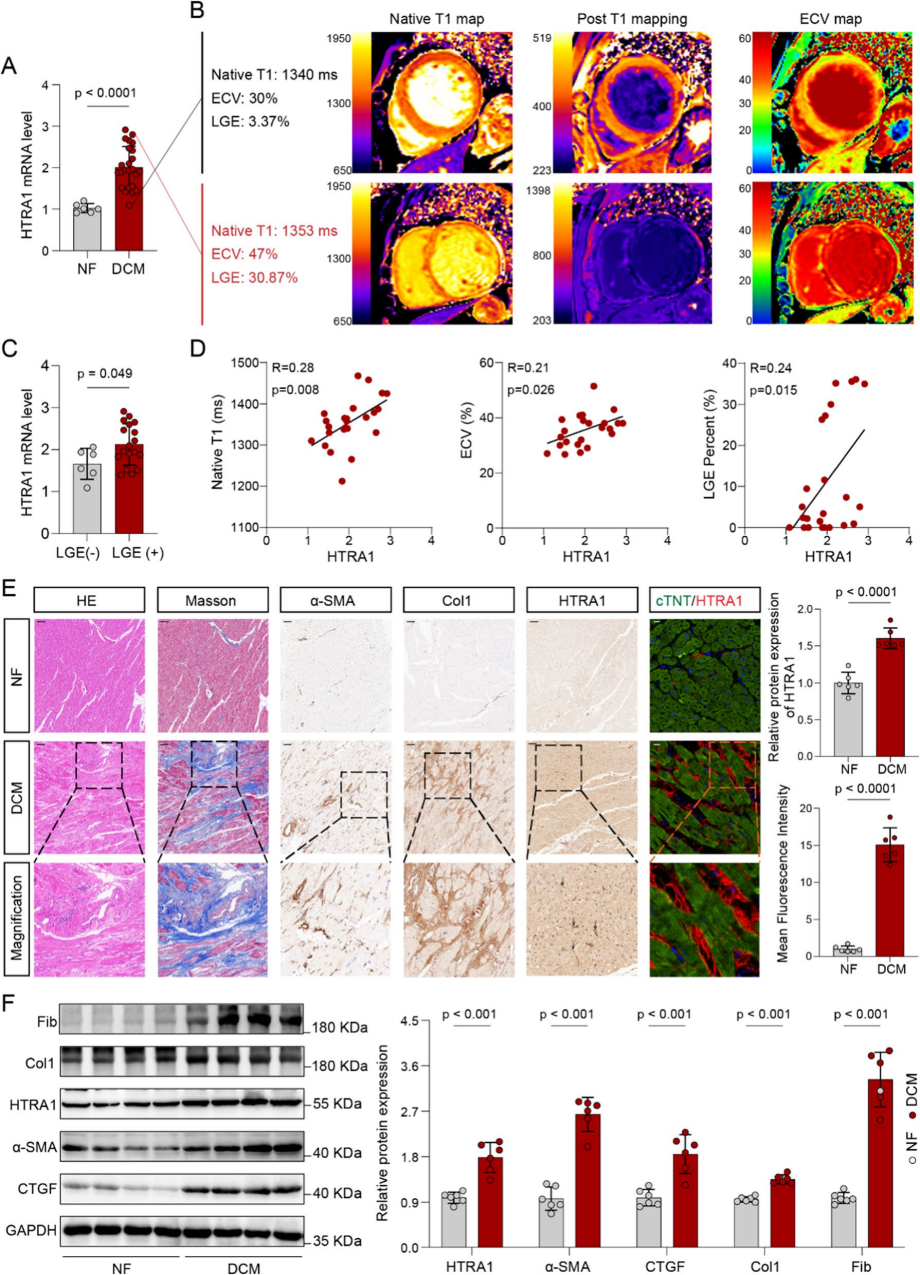
HTRA1 was upregulated in human DCM heart tissues and correlated with myocardial fibrosis. **A**. qRT-PCR assay showing the difference of HTRA1 mRNA expression between DCM and NF tissues (n: DCM:NF = 24:6). **B**. Representative cases of co-registered LV native T1 maps, ECV maps in different HTRA1 mRNA expression patients. **C** The difference of HTRA1 expression between LGE (-) and LGE ( +) patients. **D** Pearson’s chi-squared test demonstrating the relationship between HTRA1 mRNA expression and clinical myocardial fibrosis parameters, including ECV, native T1, and LGE, which were evaluated through cardiac magnetic resonance imaging. **E** Immunofluorescence and immunohistochemical assessments for HTRA1, Col1 and α-SMA in human DCM and NF tissues. Masson staining for the fibrosis abundance of tissues. The lower scale bar indicates 100um, and the higher scale bar indicates 20 μm. **F** Western blot analysis to assess the protein level difference of HTRA1 and fibrogenic proteins including α-SMA, CTGF, Col1 and Fib between DCM and NF patients

Furthermore, we conducted validation experiments on HTRA1 in human cardiac tissue. qRT-PCR analysis confirmed a notably elevated expression of HTRA1 specifically in DCM cases (Fig. 2A). Recognizing the advantages of cardiovascular magnetic resonance (CMR) imaging in assessing myocardial fibrosis, we collected imaging data from 24 DCM patients, including native T1, ECV, and LGE, for subsequent analysis. These imaging parameters have been proven to be positively correlated with the degree of myocardial fibrosis [45]. The low expression group of HTRA1 exhibited lower levels of Native T1, ECV and LGE percent (Fig. 2B, Table 1). HTRA1 level in LGE ( +) was higher than in LGE (−) (Fig. 2C). Pearson’s correlation analysis further revealed a strong positive association between HTRA1 mRNA level and cardiac fibrosis imaging markers (Fig. 2D). Immunohistochemistry, immunofluorescence, and Immunoblotting analyses consistently demonstrated a significant overexpression of HTRA1 in individuals with DCM. Remarkably, HTRA1 expression showed a strong positive correlation with the expression of fibrosis-associated molecules, including Col1 and α-SMA, as well as the level of interstitial fibrosis (Fig. 2E, F, Additional file 1: Fig. S1A). These intriguing results strongly imply the substantial involvement of HTRA1 in the progression of myocardial fibrosis in DCM, thus warranting further comprehensive investigations.

To check if HTRA1 is associated with fibrogenesis in mice with DCM, a DOX mice dataset (GSE97642) was reanalyzed. The results showed that HTRA1 was positively correlated with fibrosis indicators like Col1, α-SMA and CTGF (Additional file 1: Fig. S2A), indicating that HTRA1 is involved in fibrogenesis of DCM mice. Furthermore, DCM mice were successfully induced through injecting Dox into mice (i.p) (Additional file 1: Fig. S2B). Immunohistochemistry, immunofluorescence and western blot analysis demonstrated that HTRA1 was markedly upregulated in mice with DCM (Additional file 1: Figs. S1B, S2C, D,).

### Knockdown of HTRA1 in vivo improves Dox-induced DCM-associated myocardial fibrosis and myocardial dysfunction in vivo

**Fig. 3:**
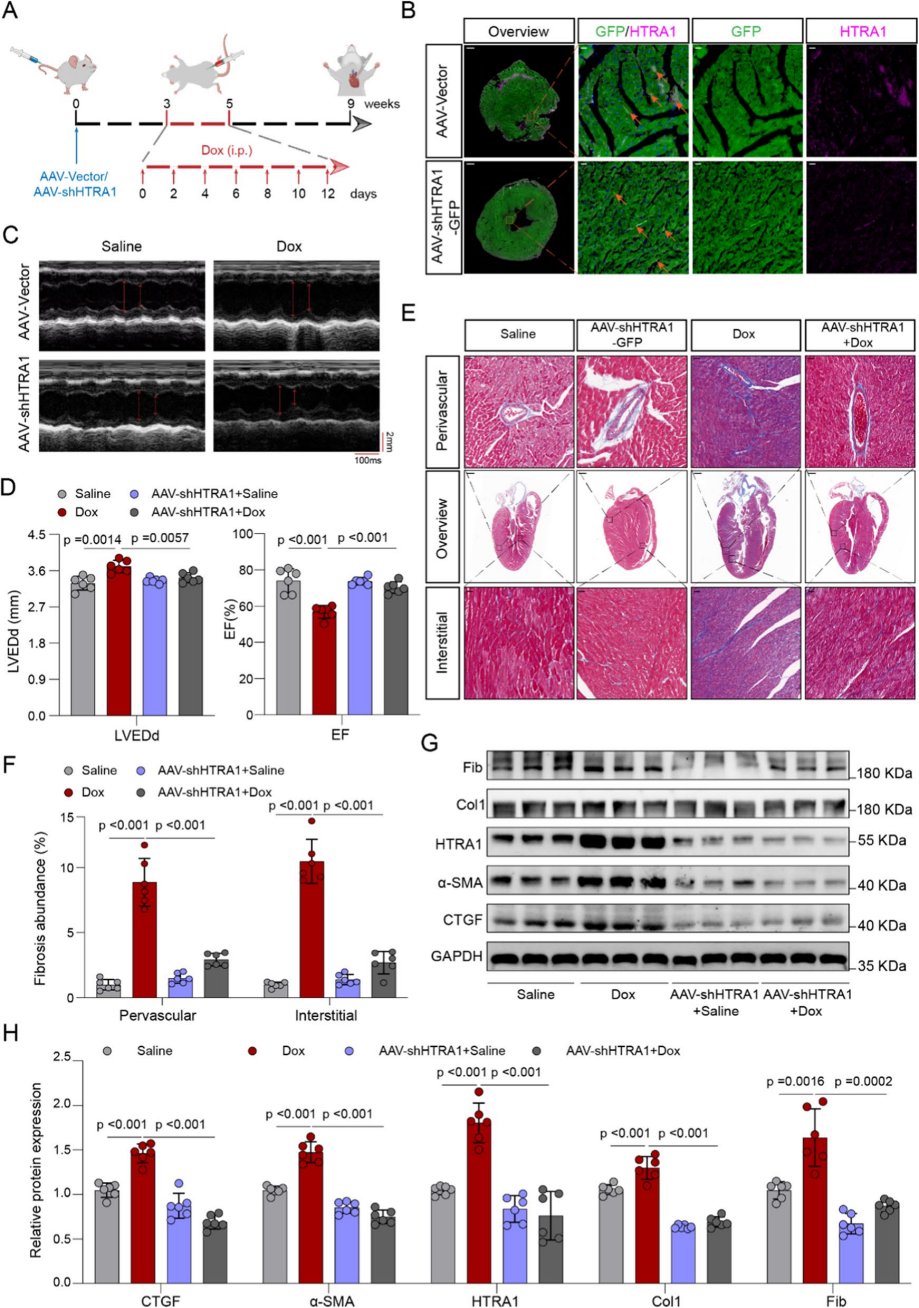
Targeted inhibition of HTRA1 can improve Dox-induced myocardial fibrosis and cardiac contractile dysfunction. **A** Animal experiment process, including AAV9 injection and Dox intervention. **B** Immunofluorescence staining of tissues showing the infection efficiency of AAV9 in mouse heart. The lower scale bar indicates 500um, and the higher scale bar indicates 20 μm. Representative echocardiography images (**C**) and quantitative data (**D**) assessing the difference of LVEDd and EF% in different groups (n = 6). Masson staining (**E**) and quantitative data (**F**) showing the fibrosis abundance in DCM mice with or without HTRA1 inhibition. The lower scale bar indicates 500um, and the higher scale bar indicates 20 μm. Fibrogenic proteins including col1, α-SMA, CTGF and Fib western blotting (**G**) and quantitative data (**H**) in DCM mice with or without HTRA1 inhibition

To investigate the potential of HTRA1 knockdown in ameliorating myocardial fibrosis in DCM mice, we utilized AAV9-shHTRA1-GFP to infect the heart tissue of C57BL/6 mice. Prior to establishing the mouse DCM model, AAV9 viruses were administered to the mice through tail vein injection for a duration of three weeks. As shown in Additional file 1: Fig. S3, AAV9-shHTRA1-GFP has the strongest infection ability to mouse heart, followed by vessels and kidneys, while its infection ability to other organs is relatively weak. All mice were sacrificed three weeks after DOX intervention (Fig. 3A). Immunofluorescence staining of the tissues demonstrated successful infection of mouse hearts by AAV9 viruses, as indicated by the presence of GFP. And HTRA1 was mainly distributed around myocardial cells (Fig. 3B). Inhibition of HTRA1 resulted in improved cardiac function in DCM mice, as evidenced by the increased EF, FS, and decreased LVEDd, and LVEDs (Fig. 3C, D, Fig. S1C). Furthermore, HTRA1 knockdown alleviated myocardial fibrosis in mice with DCM (Fig. 3E, F). Downregulation of HTRA1 inhibited the protein expression of Col1, α-SMA, Fib, and CTGF in mice with DCM (Fig. 3G, H). These findings suggest that inhibition of HTRA1 can protect against Dox-induced DCM-associated myocardial fibrosis and myocardial dysfunction in vivo.

### HTRA1 is enriched in cardiac fibroblasts and promotes the conversion of fibroblasts to myofibroblasts

**Fig. 4:**
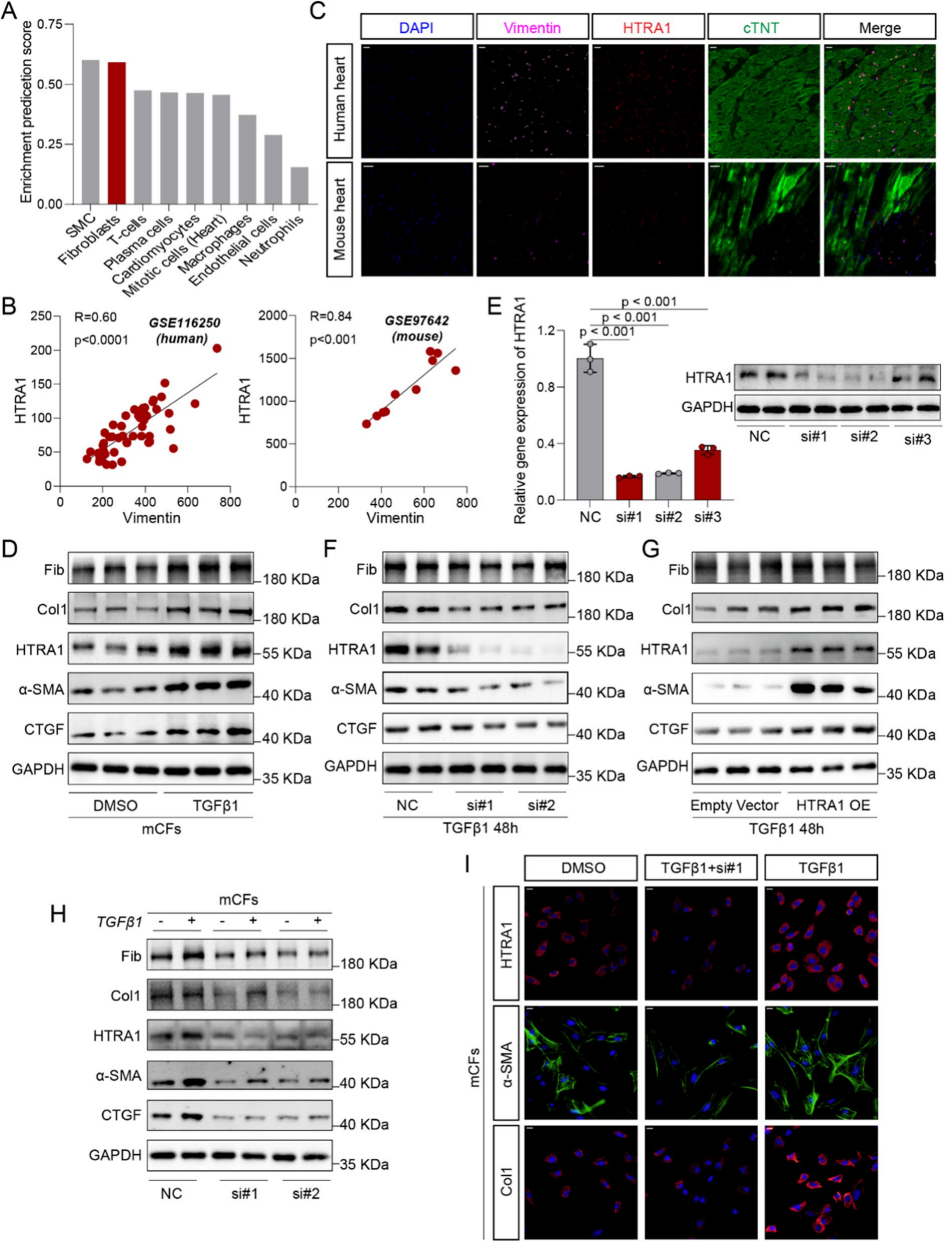
Inhibition of HTRA1 attenuated the activation of cardiac fibroblasts induced by TGFβ1. **A** The enrichment prediction score of HTRA1 in heart tissue cell types from HPA datasets (https://www.proteinatlas.org/). **B** Linear regression showing positive correlation between HTRA1 and fibroblast marker (Vimentin) in human (GSE116250, n = 51) and mouse heart tissues (GSE97642, n = 10). **C** Representative immunofluorescence images showing the colocalization of HTRA1 and cardiac fibroblast marker (Vimentin) in human and mouse heart tissues. The scale bar indicates 20 μm. **D** Representative western blot showing the change of HTRA1 protein expression in activated cardiac fibroblasts induced by TGFβ1. **E** qRT-pcr and western blot exhibiting the knockdown effect of HTRA1 siRNA. Representative western blot displaying the changes of fibrogenic proteins, including CTGF, α-SMA, Col1 and Fib, after inhibiting (**F**) or overexpressing (**G**) HTRA1. Representative immune blot (**H**) and immunofluorescence images (**I**) showing the fibrogenic proteins expression such as Col1 and α-SMA. The scale bar indicates 20 μm. Primary cardiac fibroblasts were transfected with HTRA1-siRNA, and/or treated with TGFβ1 for 48 h

According to the enrichment prediction score of HTRA1 in heart tissue cell types from the human protein atlas database (HPA, https://www.proteinatlas.org/), we found HTRA1 was mainly enriched in smooth muscle cells and cardiac fibroblasts (Fig. 4A). Pearson correlation analysis based on the human DCM (GSE116205) and Dox mice (GSE97642) datasets further revealed a strongly correlation between HTRA1 and fibroblast marker (Vimentin) (Fig. 4B). Immunofluorescence analysis indicated that HTRA1 is mainly distributed around cardiomyocyte and co-localizes selectively with fibroblasts in human and mouse heart (Fig. 4C). Thus, cardiac fibroblasts were chosen to explore the functions of HTRA1 in DCM fibrogenesis.

It has been demonstrated that TGFβ1 could induce cardiac fibroblasts to undergo phenotypic transformation into cardiac myofibroblasts and ECM synthesis. In this study, TGFβ1 was used to activate cardiac fibroblasts (20 ng/ml for 48 h). HTRA1 protein level was significantly upregulated in activated cardiac fibroblasts accompanied by the overexpression of fibrogenic proteins such as Col1, CTGF, Fib and α-SMA (Fig. 4D, Additional file 1: Fig. S1D). Then, we successfully constructed HTRA1 small interfering RNA (Fig. 4E) and overexpression plasmid to knockdown or overexpressed HTRA1 expression. After inhibiting or upregulating HTRA1 expression in primary cardiac fibroblasts, the expression of fibrogenic proteins like Col1, CTGF, Fib and α-SMA were considerably attenuated (Fig. 4F, Additional file 1: Fig. S1E) or overexpressed (Fig. 4G, Additional file 1: Fig. S1F). Furthermore, with the inhibition of HTRA1, cardiac fibroblasts could not be activated by TGFβ1 (Fig. 4H, I, Additional file 1: Fig. S1G, H).

### HTRA1 promotes the translocation of Col1 from the endoplasmic reticulum to the Golgi apparatus

**Fig. 5:**
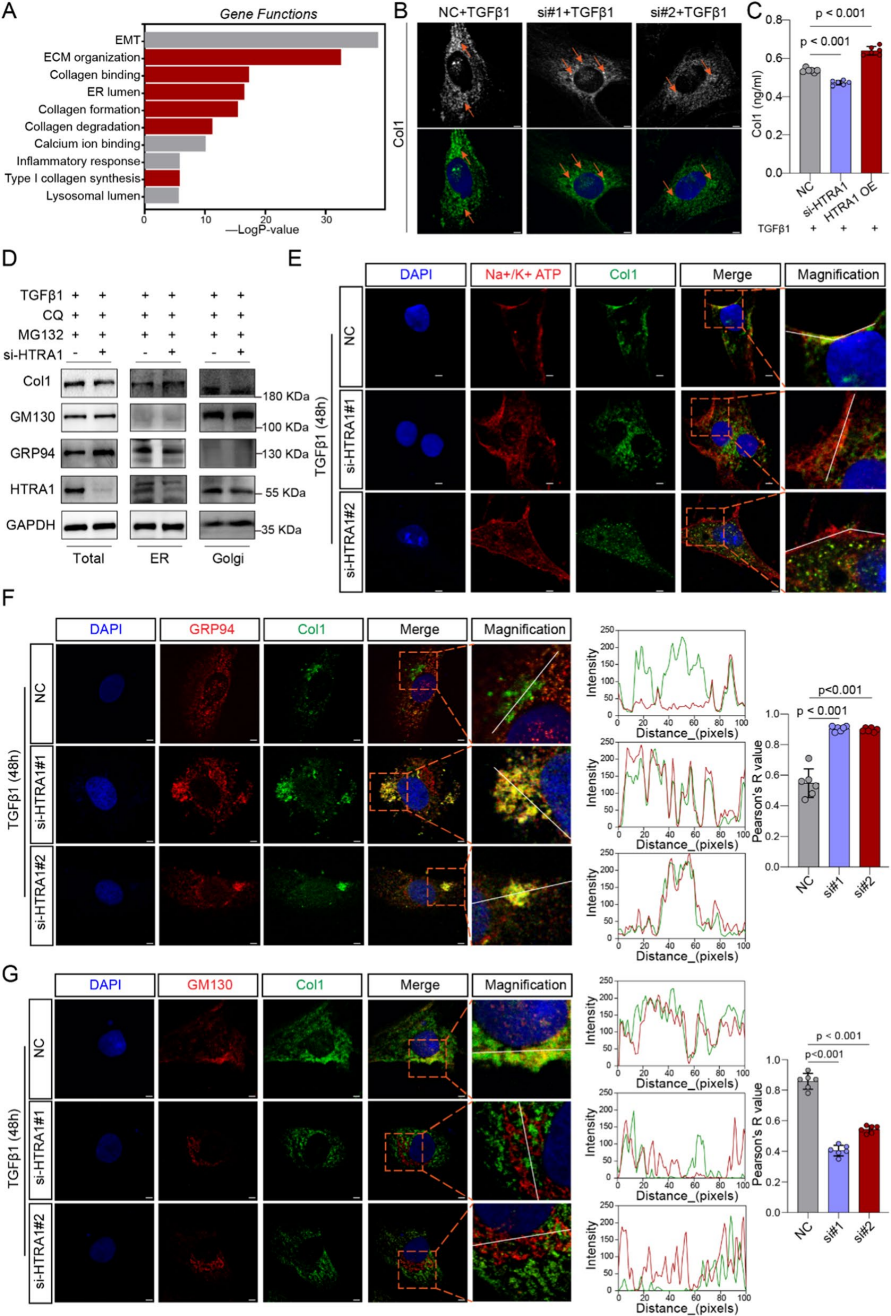
HTRA1 promoted the ER-to-Golgi transport and secretion of Col1. **A** Gene functions enrichment analysis for HTRA1. The 37 DCM samples from GSE116250 were divided into two groups based on the median expression value of HTRA1. The differentially expressed genes (|log(FC)|≥ 0.58, p < 0.05) from two groups were then imported into the Metascape database (https://metascape.org/) for enrichment analysis. **B** Representative immunofluorescence images showing the distribution of Col1 (orange arrows) in primary cardiac fibroblasts transfected with HTRA1 siRNA. The scale bar indicates 20 μm. **C** ELISA assays showing the Col1 concentration difference of cell supernatant in cardiac fibroblasts transfected with or without HTRA1 siRNA or HTRA1 plasmid. **D** Western blot showing the effect of HTRA1 inhibition on col1 protein expression in the whole cell, ER and Golgi apparatus, treated with MG132 (10 µM, 8 h) and CQ (25 µM, 8 h). **E** Representative immunofluorescence images displaying the colocalization of Col1 and Na + /K + ATP. Orange box represented a typical colocalization field of view. The scale bar indicates 4um. Immunofluorescence assay (left images) exhibiting the colocalization of col1 and GRP94 (**F**) or GM130 (**G**) in cardiac fibroblasts treated with or without HTRA1 siRNA. Orange box represented a typical colocalization field of view. The scale bar indicates 4 μm. Middle images showing the intensity of col1 and GRP94/GM130 along with the whit lines. Right box plots quantifying colocalization strength using Pearson’s R. Primary cardiac fibroblasts were treated with TGFβ1 for 48 h

To identify HTRA1 related functions, enrichment analysis of gene ontology based on Metascape database was performed and the results showed that HTRA1 was associated with ECM organization, collagen formation, type I collagen synthesis, ER lumen and lysosomal lumen (Fig. 5A). Thus, we further explored the change of Col1 localization after knocking down HTRA1. Interestingly, immunofluorescence assays demonstrated that Col1 mainly distributed in the perinuclear region in HTRA1 downregulated group (orange arrow), while in the control group, Col1 was widely distributed in the cytoplasm (orange arrow), indicating that HTRA1 could affect the distribution of Col1 in cardiac fibroblasts (Fig. 5B). Also, a significant increase/reduce in Col1 concentration in cell supernatant, which was collected from inhibiting/upregulating HTRA1 cardiac fibroblasts, was found by ELISA detection (Fig. 5C). These results convince us HTRA1 is involved in the process of Col1 formation and secretion. Considering the maturation and secretion of Col1 needs sequentially to passe through the ER and Golgi apparatus, we believe Col1 locating in the perinuclear region in HTRA1 downregulated group actually stays in ER lumen. Therefore, we respectively extracted ER and Golgi apparatus proteins from cardiac fibroblasts which was treated with negative control or HTRA1 small interfering RNA after inducing by CQ and MG132. Western blot analysis indicated depletion of HTRA1 significantly blocked Col1 proteins in the ER lumen and inhibited the Col1 protein expression in Golgi apparatus (Fig. 5D). Consistently, the absence of HTRA1 remarkably reduced the presence of Col1 in the cell membrane (Na + /K + ATP) (Fig. 5E, Additional file 1: Fig. S4) and Golgi apparatus (GM130) (Fig. 5G), while enhancing the accumulation of Col1 in the ER (GRP94) (Fig. 5F). Taken together, these results suggest HTRA1 promotes the Golgi residency and secretion of Col1.

### HTRA1 promotes the formation of ER exit sites by recruiting SEC16A, thereby accelerating the secretion of Col1

**Fig. 6:**
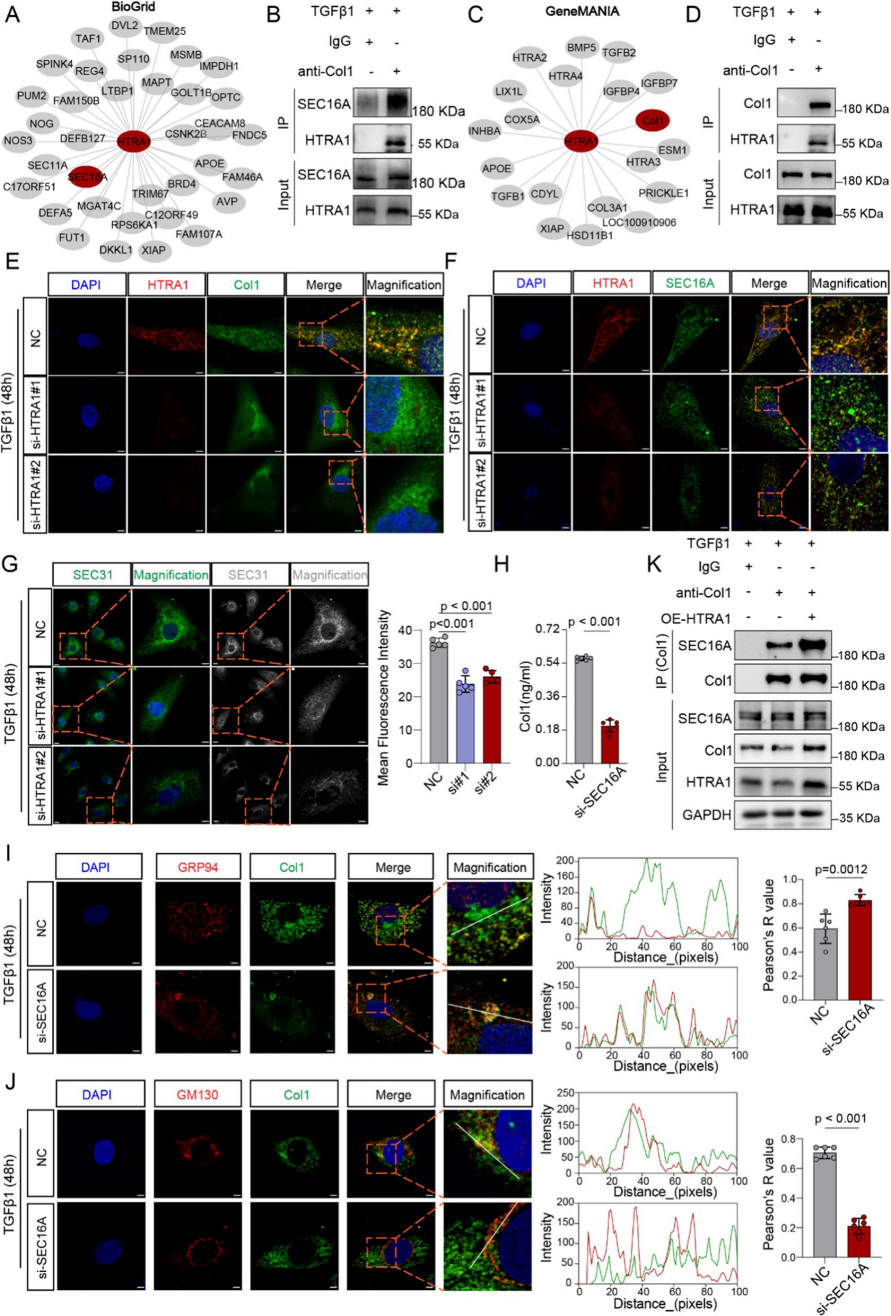
HTRA1 promoted the formation of ERES by recruiting SEC16A, which was indispensable for the ER-to-Golgi apparatus transport. **A** Display of interacting proteins with HTRA1 mined from the BioGrid database (https://thebiogrid.org/). **B** Representative Co-immunoprecipitation (Co-IP) showing the interaction between HTRA1 and SEC16A in cardiac fibroblasts. **C** Display of interacting proteins with HTRA1 mined from the GeneMANIA database (https://genemania.org/). **D** Representative Co-immunoprecipitation (Co-IP) showing the interaction between HTRA1 and Col1 in cardiac fibroblasts. Representative immunofluorescence images showing the colocalization of HTRA1 and Col1 (**E**) or SEC16A (**F**) in cardiac fibroblasts treated with or without HTRA1 siRNA. Distribution of Col1 (**E**) or SEC16A (**F**) was also exhibited. Orange box represented a typical colocalization field of view. The scale bar indicates 4 μm. (**G**). Immunofluorescence assays and quantitative data showing the changes of SEC31 (ERES marker) intensity in HTRA1 siRNA group (left). Grayscale mode can better display the intensity and distribution differences of SEC31. Orange box represented a typical colocalization field of view. The lower scale bar indicates 10 μm, and the higher scale bar indicates 4 μm. **H** ELISA assays showing the Col1 concentration difference of cell supernatant in cardiac fibroblasts transfected with or without SEC16A siRNA. Immunofluorescence assay (left images) exhibiting the colocalization of Col1 and GRP94 (**I**) or GM130 (**J**) in cardiac fibroblasts treated with or without SEC16A siRNA. The scale bar indicates 4 μm. Orange box represented a typical colocalization field of view. Middle images showing the intensity of Col1 and GRP94/GM130 along with the whit lines. Right box plots quantifying colocalization strength using Pearson’s R. **K** Co-IP analysis showing the interaction between col1 and SEC16A in cardiac fibroblasts treated with HTRA1 plasmid. Primary cardiac fibroblasts were treated with TGFβ1 for 48 h

In order to explore the key mechanisms by which HTRA1 affects the maturation and secretion of Col1, we explored the proteins interacting with HTRA1 through online databases the BioGrid and GeneMANIA. The results demonstrated that HTRA1 could interact with SEC16A and Col1, which was then successfully verified by Co-IP assays (Fig. 6A–D). In addition, depletion of HTRA1 markedly inhibited the co-localization of HTRA1 with Col1 (Fig. 6E) and SEC16A (Fig. 6F), increasing distribution of Col1 around the cell nucleus (Fig. 6E) and impeding the accumulation of SEC16A in the perinuclear region (Fig. 6F).

As we all known, COPII vesicles mediate the sorting and transport of various secretory cargo, including collagen, from the ER to the Golgi apparatus. The initiation assembly site of COPII vesicles on the ER membrane, known as the ERES, serves as a central conduit for the export of cargo from the ER which could affect the secretion of proteins [46, 47]. Recently, SEC16A has been defined as a crucial protein that is necessary for the creation of ERES and the transport of COPII vesicles [22, 48, 49]. Further analysis demonstrated that downregulation of HTRA1 indeed reduced the ERES intensity (SEC31) (Fig. 6G). To explore whether SEC16A could affect Col1 secretion, we constructed SEC16A small interfering rna to inhibit its expression in cardiac fibroblasts. ELISA assay suggested inhibition of SEC16A significantly decreased the secretion of Col1 (Fig. 6H). Consistent with our previous results (Fig. 6E, F), knocking down SEC16A strongly enhanced the accumulation of Col1 in the ER (GRP94) (Fig. 6I), while reducing the presence of Col1 in the Golgi apparatus (GM130) (Fig. 6J). Additionally, overexpression of HTRA1 enhanced interaction between Col1 and SEC16A (Fig. 6K), which led us to believe HTRA1 plays a crucial role in guiding Col1 to transport to the ERES. Taken together, these results suggested HTRA1 not only upregulated the expression of Col1, but also promoted the transport of Col1 from the ER to the Golgi apparatus through promoting the formation of ERES. Given the enrichment of fibrosis-related Col3 and TGFβ/BMPs signaling in the interaction analysis, we further validated the relationship between HTRA1 and them. The results showed that silencing HTRA1 significantly inhibited the expression of Col3 (Additional file 1: Fig. S5A), but immunofluorescence analysis did not reveal any effect of HTRA1 on the intracellular distribution of Col3 (Additional file 1: Fig. S5B, C). Knocking down HTRA1 did not affect the expression of downstream molecules p-SMAD5/SMAD5 and p-SMAD2/SMAD2 of TGFβ/BMPs (Additional file 1: Fig. S5A), suggesting that HTRA1 may not affect the activity of the TGFβ/BMPs signaling pathway in cardiac fibroblasts.

### HTRA1 inhibition propelled the degradation of remaining Col1 in ER lumen by inducing ER- autophagy

**Fig. 7:**
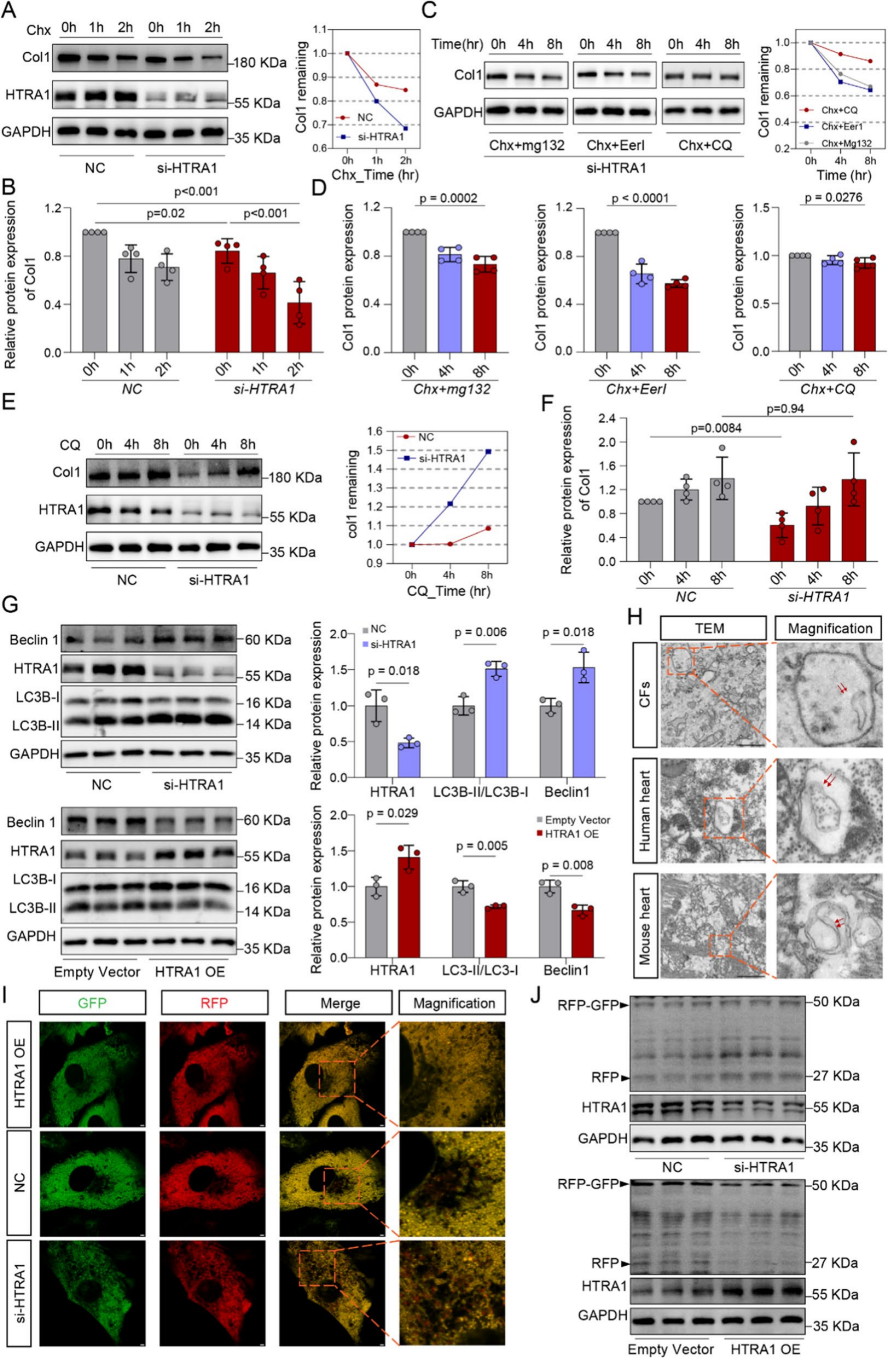
Inhibition of HTRA1 promoted the endoplasmic reticulum autophagy degradation of Col1. Western blot (**A**) and quantitative data (**B**) showing the trends of col1 protein expression under different Chx treatment time (0 h, 1 h, 2 h) in cardiac fibroblasts transfected with or without HTRA1 siRNA. Representative Western blot (**C**) and quantitative data (**D**) showing the change of Col1 protein expression under different treatments including Chx + MG132, Chx + EerI and Chx + CQ in cardiac fibroblasts transfected with HTRA1 siRNA. Cardiac fibroblasts were respectively treated for 0, 4 and 8 h. Representative Western blot (**E**) and quantitative data (**F**) showing the change of Col1 protein expression under different CQ treatment time (0 h, 4 h, 8 h) in cardiac fibroblasts transfected with or without HTRA1 siRNA. **G** Representative western blot and quantitative data showing the protein expression difference of autophagy indicators including Beclin1 and LC3B between negative control (NC) and HTRA1 siRNA or HTRA1 plasmid group. Grey represented the control group, blue represented the HTRA1 siRNA group, and red represented the HTRA1 plasma group. **H** Representative electron microscopy showing endoplasmic reticulum autophagosomes or autophagic lysosomes in cardiac fibroblasts, human and mouse heart. The scale bar indicates 1um. Orange box represented a typical colocalization field of view. Red arrow refers to endoplasmic reticulum segments. **I** Live-cardiac fibroblasts imaging transfected with the ssRFP-GFP-KDEL tandem reporter plasmid for 24 h. The scale bar indicates 2 μm. Counting the number of red dots in cells for evaluating the intensity of endoplasmic reticulum autophagy. Twenty cells in every group were counted. **J** The band intensities of RFP and RFP-GFP were determined, and the normalized RFP:RFP-GFP ratio was presented

Since the gene functions enrichment analysis showed a significant correlation between HTRA1 and collagen degradation (Fig. 5A), and downregulation of HTRA1 obviously accelerated the exhaustion of Col1 (Fig. 7A, B), we were attempting to explore how HTRA1 regulates the degradation of Col1. After silencing HTRA1 and suppressing protein synthesis with cycloheximide (CHX), cardiac fibroblasts were respectively treated with proteasomal inhibitor MG-132, endoplasmic reticulum-associated degradation (ERAD) inhibitor eeyarestatin I (Eer I) and lysosomal inhibitor chloroquine (CQ). The results indicated that only CQ could markedly delay the degradation of Col1 (Fig. 7C, D). Comparing with negative control group, HTRA1 depletion group had slower Col1 degradation rate after treating with CQ (Fig. 7E, F), which suggested Col1 remaining in ER lumen is mainly degraded by lysosomes. As we all known, the degradation of autophagy cargo and the recycling of products (such as amino acids) by lysosomes are the final steps of autophagy [50]. Recently, in addition to ERAD pathways, ER autophagy has been identified as a new protein degradation mechanism, which can degrade part of ER and proteins remaining in ER lumen to maintain the homeostasis of ER quality [31]. Based on our findings, we hypothesize that the suppression of HTRA1 in cardiac fibroblasts activates ER autophagy, thereby facilitating the degradation of Col1. As expected, knocking down HTRA1 significantly increased LC3B-II/LC3B-I and Becline-1 protein expression, while overexpression of HTRA1 markedly reduced their expression (Fig. 7G). ER autophagy, characterized by autophagosomes containing fragments of the ER under electron microscopy, was observed in cardiac fibroblasts, human and mouse heart respectively (Fig. 7H). In addition, to quantitatively evaluate the impact of changes in HTRA1 expression on ER autophagy, the previously described ssRFP-GFP-KDEL sensor was constructed (ss: ER signal sequence; KDEL: Endoplasmic reticulum retention sequence) [51]. Under normal conditions, ssRFP-GFP-KDEL is localized in the ER. However, during ER autophagy, it could enter the lysosome, where ssRFP-GFP-KDEL can be cleaved by lysosomal enzymes into ssRFP and GFP-KDEL fragments. Under acidic conditions within the lysosome, GFP fluorescence rapidly quenches, while the fluorescence from ss-RFP remains stable. Therefore, the degree of ER autophagy can be assessed by counting the number of red puncta or by measuring the relative expression level of RFP protein using Western blot analysis. Compared with the negative control group, knocking down HTRA1 significantly increased the number of red spots and RFP/RFP-GFP ratio, while overexpressing HTRA1 reduced the number of red spots and RFP/RFP-GFP ratio (Fig. 7I, J, Additional file 1: Fig. S6A, B). To sum up, in the HTRA1-depleted group, the degradation of residual Col1 in the ER lumen is mediated by HTRA1 knockdown-induced ER-autophagy.

### Inhibiting HTRA1 induces ER autophagy by activating ER stress

**Fig. 8:**
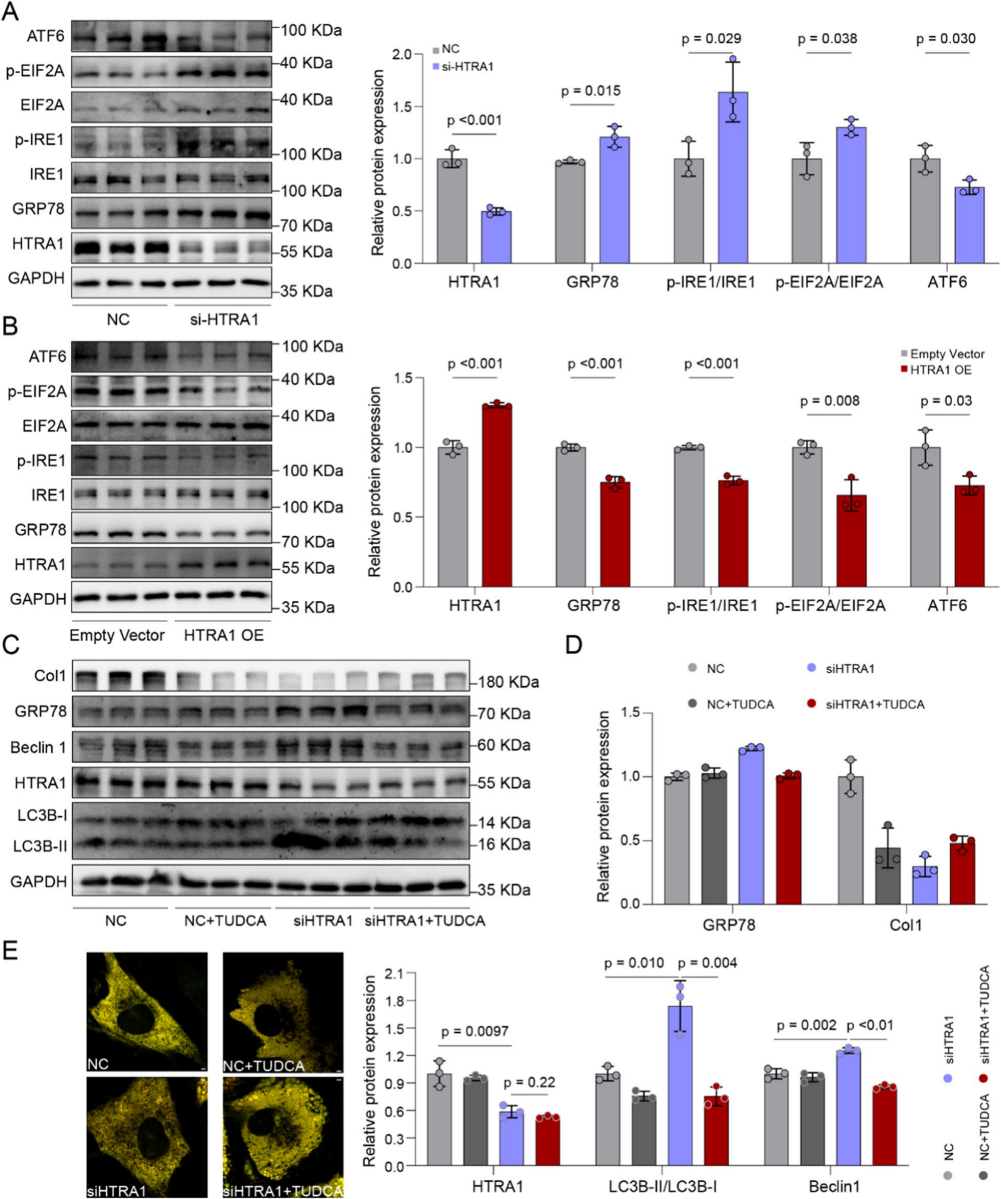
ER stress induced by inhibition of HTRA1 activated ER autophagy. ER stress indicators like GRP78, ATF6, p-EIF2A and p-IRE1 western blotting and quantitative data in cardiac fibroblasts transfected with HTRA1 siRNA (**A**) or HTRA1 plasmid (**B**). Grey represented the control group, blue represented the HTRA1 siRNA group, and red represented the HTRA1 plasma group. Representative western blotting (**C**) and quantitative data (**D**) showing the effect of broad ER stress inhibitor tauroursodeoxycholic acid (TUDCA, 0.5 mmol/l, 24 h) on ER autophagy in cardiac fibroblasts transfected with HTRA1 siRNA. GRP78, Beclin1 and LC3B were detected. (**E**). Live-cardiac fibroblasts imaging in different groups transfected with the ssRFP-GFP-KDEL tandem reporter plasmid for 24 h. The scale bar indicates 2 μm

ER stress is an intracellular adaptive regulatory biological process characterized primarily by the accumulation of misfolded and unfolded proteins within the cell [52]. Previous studies have revealed complex interactions between ER stress and autophagy, where activation of ER stress can promote autophagy, further contributing to the progression of cardiovascular diseases [32]. Here, we have confirmed that inhibiting HTRA1 can promote the retention of Col1 in the ER, undoubtedly increasing the burden on the ER and triggering the activation of ER stress. As we expected, GRP78, an ER stress marker, was significantly increased in HTRA1-inhibition group and downregulated in HTRA1 overexpression group (Fig. 8A, B). To investigate whether ER stress induced by HTRA1 knockdown can activate ER autophagy, we employed a broad-spectrum ER stress inhibitor, Tauroursodeoxycholic acid (TUDCA), to intervene in cardiac fibroblasts following HTRA1 knockdown. The results demonstrated that the broad-spectrum ER stress inhibitor significantly restored ER autophagy triggered by HTRA1 knockdown, while there was a mild upregulation of Col1 expression (Fig. 8C–E). These findings suggest that the ER stress caused by the retention of Col1 in the ER lumen in HTRA1 inhibition group, can activate ER autophagy, facilitating the lysosomal degradation of Col1 and thereby maintaining ER homeostasis.

Additionally, when endoplasmic reticulum stress is induced by appropriate stimuli, cells can undergo unfolded protein response (UPR) to promote cell survival. However, when the stimulus is too strong and lasts for a long time, cells initiate apoptosis programs [53, 54]. After knocking down HTRA1, we detected the apoptosis of cardiac fibroblasts, and the results showed that HTRA1 silencing slightly upregulated the expression of BAX protein and downregulated the expression of BCL2 (Additional file 1: Fig. S7A), which meant that inhibiting HTRA1 promoted the apoptosis of cardiac fibroblasts to some extent. Similarly, a slight increase in the apoptosis level of cardiac fibroblasts (Tunel) was observed in the DCM model constructed by AAV-shHTRA1 knockdown mice (Additional file 1: Fig. S7B). These results suggested that endoplasmic reticulum stress caused by HTRA1 silencing may trigger apoptosis.

## Discussion

Herein, we identified HTRA1 as a crucial selective regulator of Col1 maturation and secretion. Inhibition of HTRA1 expression considerably promotes ER stress-induced ER autophagy-mediated Col1 degradation. The findings of this study provide valuable insights regarding the therapeutic potential of targeting HTRA1-mediated collagen secretion for treating myocardial fibrosis in DCM.

**Fig. 9:**
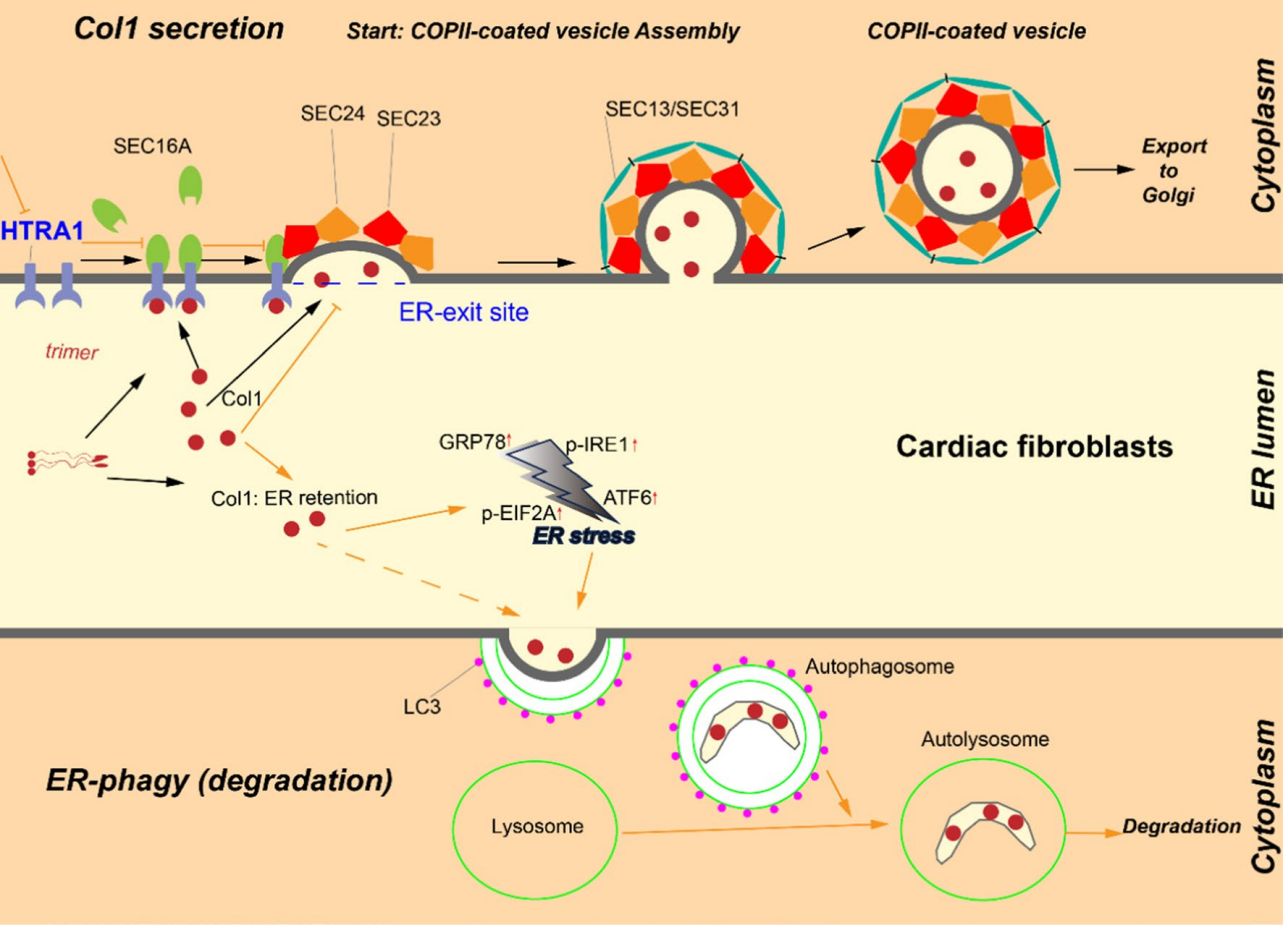
Mechanism diagram. In DCM, increased HTRA1 levels drive fibroblast-to-myofibroblast transition and recruite SEC16A for efficient ERES assembly. This enables myofibroblasts to rapidly secrete Col1, promoting fibrosis progression. Targeting HTRA1 results in Col1 accumulation in the ER, triggering ER stress-induced autophagy and facilitating Col1 degradation while maintaining ER stability

Collagen, a vital component of ECM, has been extensively studied for its unique physical, biochemical, and mechanical properties. It plays a critical role in various cellular processes such as cell proliferation, differentiation, and migration [55, 56]. Its significance extends to organ fibrosis, tumor development, and growth and development [57–59]. Although researchers have systematically investigated the synthesis, processing, modification, and degradation of collagen, it is only in the last two decades that novel molecules involved in collagen synthesis and transport have gradually been discovered and characterized [21, 60, 61]. However, the mechanisms underlying collagen transport from the ER to the Golgi apparatus remain poorly understood. Herein, we identified HTRA1 as a newly discovered cargo receptor responsible for the ER-to-Golgi transport of procollagen. HTRA1 plays a dual role by recruiting SEC16A to facilitate ERES assembly and acting as a specific intermediary between Col1 and ERES. This function enables the transportation of Col1 into the Golgi apparatus, thereby ensuring its proper secretion. The inhibition of HTRA1 disrupts ERES assembly and impedes the interaction of Col1 with ERES, accumulating Col1 within the ER lumen. Abnormal retention of Col1 triggers ER stress, which induces ER autophagy and subsequent selective degradation of Col1 by autolysosomes, thereby preserving ER homeostasis (Fig. 9).

SEC16A is a key molecule involved in the assembly and maintenance of ERES, and its intracellular expression and distribution vary depending on the cargo load of different secretory proteins [20]. It has been found that in Hela cells, TANGO1 can recruit SEC16A to the ER membrane, facilitating ERES assembly and ensuring efficient secretion of collagen [62]. On the other hand, TFG can expand the structure of ERES to facilitate the transport of large-molecular-weight collagen [61]. During myocardial fibrosis, it is crucial to explore the molecular mechanisms involved in the rapid recruiting of SEC16A, initiating ERES assembly, and meeting the high demand for collagen secretion. Here, we discovered that HTRA1, which is highly expressed in cardiac fibroblasts, can recruit more SEC16A proteins to the ER membrane, thereby expanding the ERES assembly. Additionally, HTRA1 selectively mediates the binding between Col1 and SEC16A, promoting ER-to-Golgi transport and the secretion of Col1. The dual role of HTRA1 in the ER provides new insights into the efficiency and accuracy of Col1 secretion, synthesis, and transport. Given that Col3 also plays an important role in organ fibrosis, we explored whether the above mechanism applies to the transport and secretion of Col3. It was found that HTRA1 does not affect the intracellular distribution of Col3. Previous studies have suggested that Col1 has a diameter of 150–300 nm, while Col3 has a diameter of 25–100 nm. COPII vesicles (with a diameter of approximately 60–80 nm) [63, 64] can accommodate the secretory demands of Col3, while the secretion of Col1 requires precise mechanisms to regulate the expansion of COPII vesicles to accommodate its transport needs. This suggests that the endoplasmic reticulum-to-Golgi transport mechanism of Col3 may differ from that of Col1. Our study found that HTRA1 can promote the assembly and expansion of endoplasmic reticulum exit sites by recruiting SEC16A to meet the transport needs of Col1. This mechanism may not apply to Col3, so the silencing of HTRA1 did not affect the intracellular distribution of Col3. The transport and secretion mechanism of Col3 requires further exploration.

When collagen secretion is imbalanced, activation of precise degradation regulatory mechanisms is particularly important for maintaining cellular homeostasis. Typically, collagen degradation pathways can be divided into intracellular and extracellular degradation, involving the degradation of newly synthesized collagen in the cytoplasm and highly crosslinked stable collagen in the ECM [65]. The extracellular degradation of collagen is mainly executed by matrix metalloproteinases (MMP) and tissue proteases, and the related mechanisms have been extensively studied. Several collagen-degrading proteases have been identified, including MMP1, MMP2, and MMP9 [66, 67], and the cysteine protease cathepsin K [68]. The intracellular degradation of collagen is primarily mediated by macropinocytosis and receptor-mediated endosomal uptake. The final step in both pathways is the fusion with lysosomes for degradation of the collagen [69, 70]. However, it remains unknown how improperly transported or unfolded Col1 within the ER is guided for degradation in lysosomes. This study revealed that the absence of HTRA1 promotes lysosomal degradation of Col1. HTRA1 inhibition leads to the abnormal retention of Col1 within the ER lumen, triggering ER stress, and subsequently activating ER autophagy. This selective autophagy promotes the lysosomal degradation of residual ER Col1 while maintaining ER homeostasis, thereby ensuring the stability of cardiac fibroblasts. Interestingly, Ishida et al. has found that the misfolded Col1, accumulating as trimers, is eliminated via the autophagy-lysosomal pathway rather than the ER-associated degradation (ERAD) pathway [15]. This is consistent with our research findings. In addition, we found that silencing HTRA1 mildly promoted the apoptosis of cardiac fibroblasts, which may be due to the inability of HTRA1-activated ER autophagy to balance the ER homeostasis disturbance caused by persistent ER stress. The apoptosis of cardiac fibroblasts in DCM can improve the level of myocardial fibrosis to some extent [71], and inhibiting HTRA1 will have a beneficial effect.

The present study defines HTRA1 as a key protease involved in ECM remodeling. It digests the active fragments produced by the ECM, which can activate fibroblasts, induce ECM remodeling, and promote disease progression [38, 72]. Studies have shown that HTRA1 can promote transforming growth factor (TGF)β1 signaling by cleaving latent TGFβ-binding protein, which directly contributes to the occurrence of cerebral small vessel disease and scar activation [38, 73]. Although these studies strongly indicate the relevance of HTRA1 in organ fibrosis, its specific functions and molecular mechanisms in organ fibrosis remain unclear. We identified HTRA1 as a critical regulatory factor in the synthesis, secretion, and degradation of Col1. This indicates a notable positive correlation between HTRA1 and ECV, LGE, native T1, and Col1 expression in patients with DCM. Targeted inhibition of HTRA1 in cardiac fibroblasts specifically promotes the lysosomal degradation of Col1, which triggers a new round of quality control in the ER and assists in the maintenance of cellular homeostasis. Because HTRA1-mediated collagen degradation occurs directly within the cells, it prevents the activation of fibroblasts by collagen fragments. Therefore, we consider HTRA1 a safe and ideal therapeutic target for treating DCM-associated myocardial fibrosis.

In summary, our study provides new insights into the precise molecular mechanisms underlying Col1 synthesis and secretion. We found that HTRA1 plays a crucial role in regulating collagen transport and secretion by recruiting SEC16A to promote ERES formation. Furthermore, our findings strongly suggest that targeting the rate-limiting steps in collagen secretion, such as HTRA1-mediated trafficking, can effectively alleviate DCM-associated myocardial fibrosis and improve cardiac function. This offers a promising therapeutic strategy for treating DCM-associated myocardial fibrosis. Future research should be focused on investigating the specific signaling pathways and molecular interactions involved in HTRA1-mediated collagen secretion. Additionally, it would be valuable to explore the potential therapeutic effects of targeting HTRA1 in other fibrotic diseases and assess its safety.

## Limitation

Although this study extensively investigated the molecular mechanisms of HTRA1 in myocardial fibrosis, some limitations remain. We did not establish HTRA1-specific knockout mice for cardiac fibroblasts, which cannot completely rule out the possibility of the involvement of HTRA1 in other cardiac cells affecting the progression of DCM-associated myocardial fibrosis. Further, as shown by the different HTRA1 scores in various cardiac cells in the Human Protein Atlas database, further investigation of the top-ranked cardiac smooth muscle cells is warranted. Additionally, the specific molecular mechanisms by which HTRA1 deficiency promotes ER stress-induced ER autophagy require further investigation. Our future research will involve the construction of conditional HTRA1 knockout mice and focus on elucidating the molecular mechanisms of HTRA1 regarding ER autophagy.

### Supplementary Information

**Additional file 1: Fig. S1.** Quantitative data plots. **A** Quantitative data of masson staining for the fibrosis abundance of tissues and quantitative data of immunohistochemical assessments for HTRA1, Col1 and α-SMA in human DCM and normal heart tissues. **B** Quantitative data of masson staining for the fibrosis abundance of tissues and quantitative data of immunohistochemical assessments for HTRA1, Col1 and α-SMA in mice heart treated with Dox and saline. **C** Quantitative data assessing the difference of LVEDs and FS% in different groups (n = 6). **D** Quantitative data of western blot showing the change of HTRA1 protein expression in activated cardiac fibroblasts induced by TGFβ1. Quantitative data of western blot displaying the changes of fibrogenic proteins, including CTGF, α-SMA, Col1 and Fib, after inhibiting (**E**) or overexpressing (**F**) HTRA1. Quantitative data of immune blot (**G**) and immunofluorescence images (**H**) showing the fibrogenic proteins expression such as Col1 and α-SMA. Primary cardiac fibroblasts were transfected with HTRA1-siRNA, and/or treated with TGFβ1 for 48h. **Fig. S2.** HTRA1 was overexpressed in Dox mice and correlated with myocardial fibrosis. **A** Scatter plots showing the correlation between HTRA1 and fibrogenic genes (Col1, α-SMA, Fib and CTGF) in Dox mice (GSE97642, n: Dox:control = 5:5; Pearson’s chi-squared test was performed). **B** Representative echocardiography images of DCM or sham mice and the assessment of echocardiography parameters including LVEDd, LVEDs, EF% and FS% (n = 6). **C** Immunofluorescence and immunohistochemical assessments for HTRA1, Col1 and α-SMA in DCM and sham mice heart tissues. Masson staining for the fibrosis abundance of tissues. The lower scale bar indicates 50 μm, and the higher scale bar indicates 20 μm. **D** Representative western blot showing the protein expression difference of HTRA1 and fibrogenic proteins including α-SMA, CTGF, Col1 and Fib between DCM and sham mice heart tissues. **Fig. S3.** Frozen section staining of heart, liver, spleen, lung, kidney and aorta after injecting AAV9-shHTRA1-GFP. **Fig. S4.** Quantitative analysis of colocalization of Col1 and Na+/K+ ATP fluorescence. **Fig. S5.** **A** Representative western blot showing the change of Col3 and p-SMAD2/5 protein expression after HTRA1 inibition. Immunofluorescence assay exhibiting the colocalization of col1 and GRP94 (**B**) or GM130 (**C**) in cardiac fibroblasts treated with or without HTRA1 siRNA. Orange box represented a typical colocalization field of view. The scale bar indicates 4 μm. Middle images showing the intensity of col1 and GRP94/GM130 along with the whit lines. **Fig. S6.** Quantitative data plots. **A** Quantitative data of red spots in different live-cardiac fibroblasts images. **B** Quantitative data showing the difference of RFP/RFP-GFP between NC and si-HTRA1 or HTRA1 plasmid groups. **Fig. S7.** Representative western blot showing the change of BCL2 and BAX protein expression after HTRA1 inhibition. **B** TUNEL staining of mouse heart tissue. The scale bar indicates 20 μm. **Table S1.** Data sets used in this study. **Table S2.** qPCR primer sequences. **Table S3.** Antibody information.

## Data Availability

All data generated in this study are available on reasonable request from the corresponding author.

## Abbreviations

DCM: Dilated cardiomyopathy
ECV: Extracellular volume fraction
LGE: Late gadolinium enhancement
ER: Endoplasmic reticulum
ERES: Endoplasmic reticulum exit site
ERAD: ER-associated degradation
DOX: Doxorubicin
MMP: Matrix metalloproteinases
