# Supplementary material for: HTRA1-driven detachment of type I collagen from endoplasmic reticulum contributes to myocardial fibrosis in dilated cardiomyopathy
Source: J Transl Med. 2024 Mar 22;22:297. doi: 10.1186/s12967-024-05098-7 (PMC10958933; doi:10.1186/s12967-024-05098-7)
Supplement: Supplementary file 1 — Additional file 1: Fig. S1. Quantitative data plots. A Quantitative data of masson staining for the fibrosis abundance of tissues and quantitative data of immunohistochemical assessments for HTRA1, Col1 and α-SMA in human DCM and normal heart tissues. B Quantitative data of masson staining for the fibrosis abundance of tissues and quantitative data of immunohistochemical assessments for HTRA1, Col1 and α-SMA in mice heart treated with Dox and saline. C Quantitative data assessing the difference of LVEDs and FS% in different groups (n = 6). D Quantitative data of western blot showing the change of HTRA1 protein expression in activated cardiac fibroblasts induced by TGFβ1. Quantitative data of western blot displaying the changes of fibrogenic proteins, including CTGF, α-SMA, Col1 and Fib, after inhibiting (E) or overexpressing (F) HTRA1. Quantitative data of immune blot (G) and immunofluorescence images (H) showing the fibrogenic proteins expression such as Col1 and α-SMA. Primary cardiac fibroblasts were transfected with HTRA1-siRNA, and/or treated with TGFβ1 for 48h. Fig. S2. HTRA1 was overexpressed in Dox mice and correlated with myocardial fibrosis. A Scatter plots showing the correlation between HTRA1 and fibrogenic genes (Col1, α-SMA, Fib and CTGF) in Dox mice (GSE97642, n: Dox:control = 5:5; Pearson’s chi-squared test was performed). B Representative echocardiography images of DCM or sham mice and the assessment of echocardiography parameters including LVEDd, LVEDs, EF% and FS% (n = 6). C Immunofluorescence and immunohistochemical assessments for HTRA1, Col1 and α-SMA in DCM and sham mice heart tissues. Masson staining for the fibrosis abundance of tissues. The lower scale bar indicates 50 μm, and the higher scale bar indicates 20 μm. D Representative western blot showing the protein expression difference of HTRA1 and fibrogenic proteins including α-SMA, CTGF, Col1 and Fib between DCM and sham mice heart tissues. Fig. S3. Frozen section staining of heart, liver [file 12967_2024_5098_MOESM1_ESM.docx]

**Additional file**

**
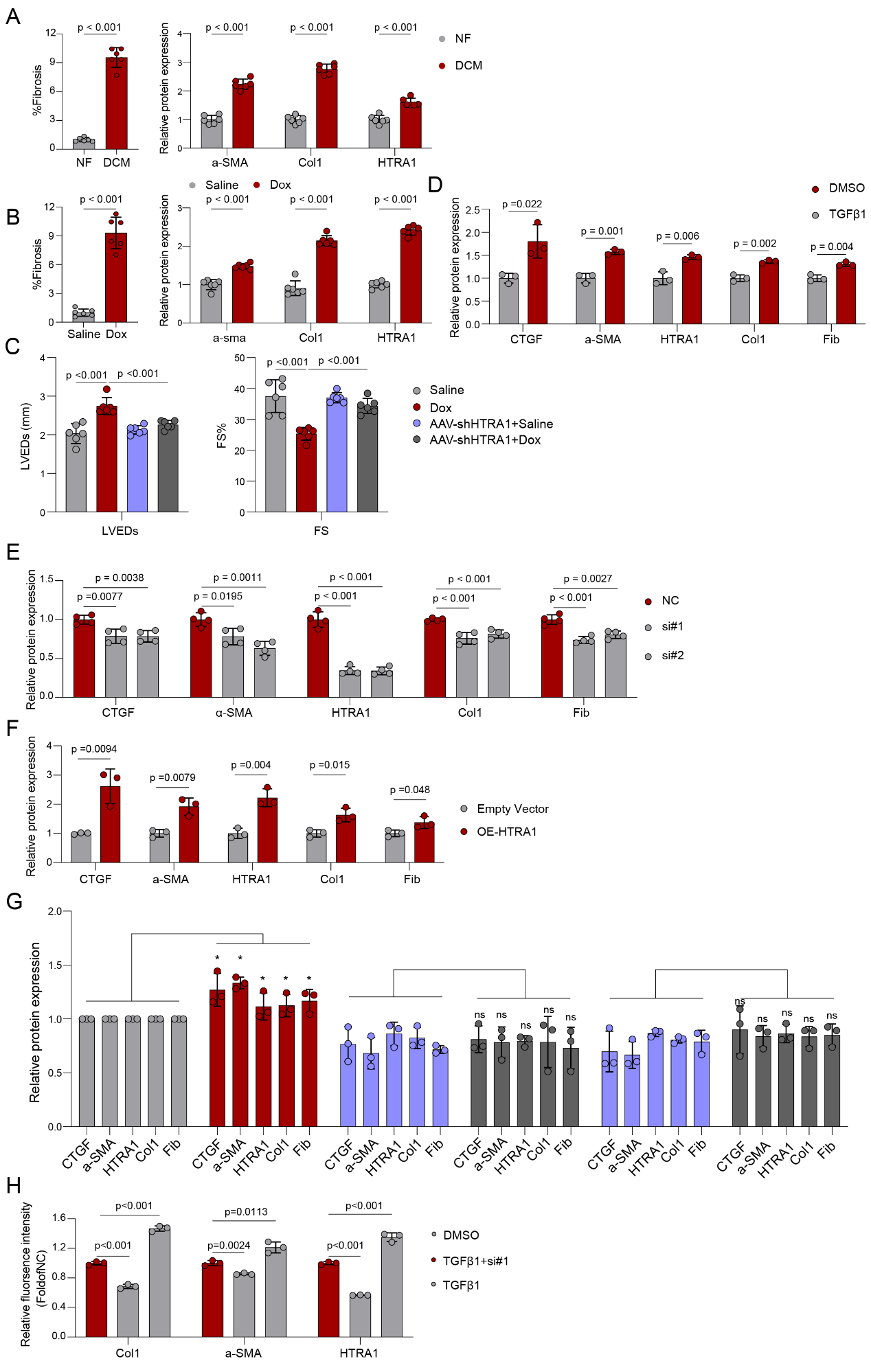
**

**Fig. S1. Quantitative data plots.**

(A). Quantitative data of masson staining for the fibrosis abundance of tissues and quantitative data of immunohistochemical assessments for HTRA1, Col1 and α-SMA in human DCM and normal heart tissues. (B). Quantitative data of masson staining for the fibrosis abundance of tissues and quantitative data of immunohistochemical assessments for HTRA1, Col1 and α-SMA in mice heart treated with Dox and saline. (C). Quantitative data assessing the difference of LVEDs and FS% in different groups (n=6). (D). Quantitative data of western blot showing the change of HTRA1 protein expression in activated cardiac fibroblasts induced by TGFβ1. Quantitative data of western blot displaying the changes of fibrogenic proteins, including CTGF, α-SMA, Col1 and Fib, after inhibiting (E) or overexpressing (F) HTRA1. Quantitative data of immune blot (G) and immunofluorescence images (H) showing the fibrogenic proteins expression such as Col1 and α-SMA. Primary cardiac fibroblasts were transfected with HTRA1-siRNA, and/or treated with TGFβ1 for 48h.

**
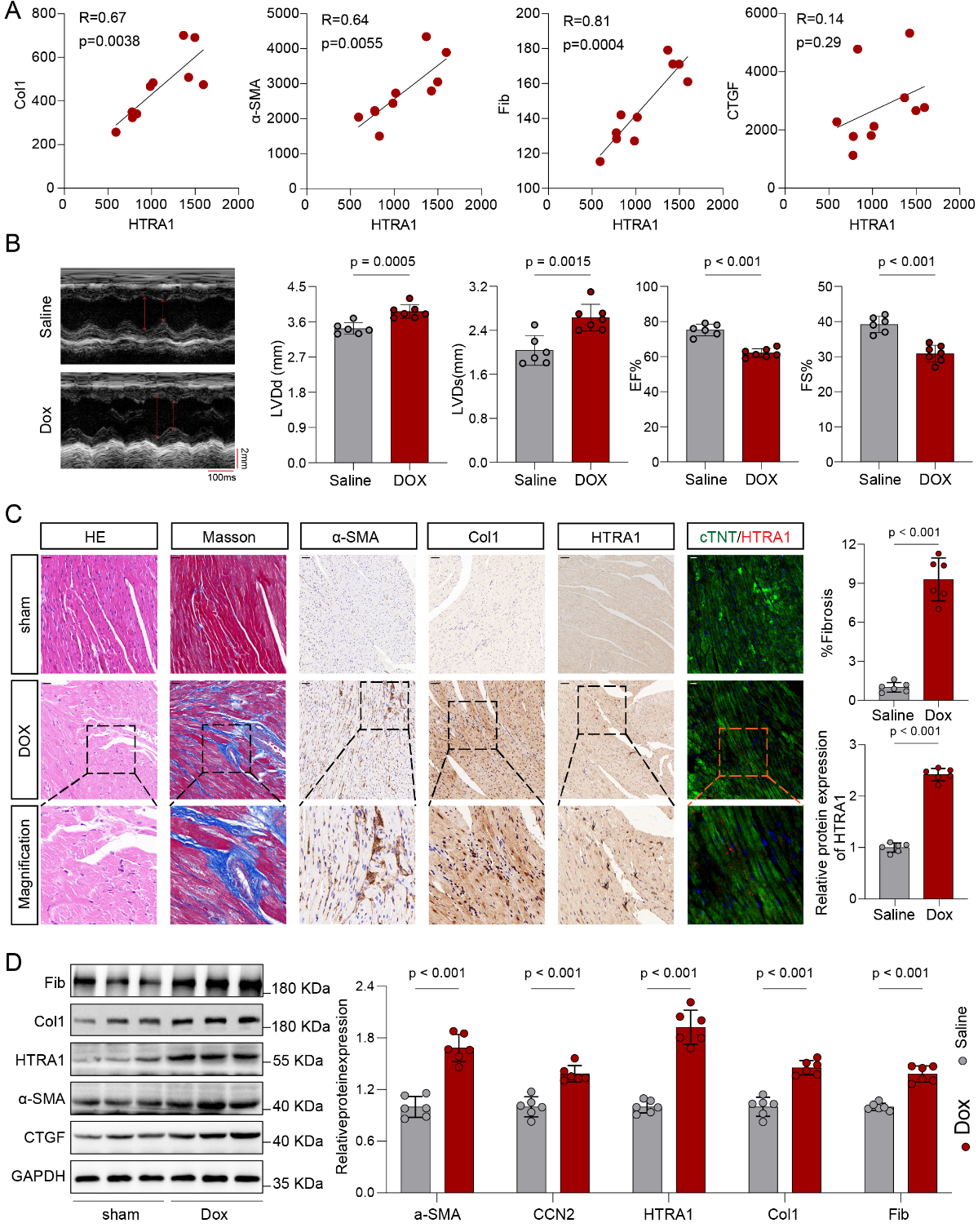
**

**Fig. S2. HTRA1 was overexpressed in Dox mice and correlated with myocardial fibrosis.**

(A). Scatter plots showing the correlation between HTRA1 and fibrogenic genes (Col1, α-SMA, Fib and CTGF) in Dox mice (GSE97642, n: Dox:control=5:5; Pearson's chi-squared test was performed). (B). Representative echocardiography images of DCM or sham mice and the assessment of echocardiography parameters including LVEDd, LVEDs, EF% and FS% (n=6). (C). Immunofluorescence and immunohistochemical assessments for HTRA1, Col1 and α-SMA in DCM and sham mice heart tissues. Masson staining for the fibrosis abundance of tissues. The lower scale bar indicates 50um, and the higher scale bar indicates 20μm. (D). Representative western blot showing the protein expression difference of HTRA1 and fibrogenic proteins including α-SMA, CTGF, Col1 and Fib between DCM and sham mice heart tissues.


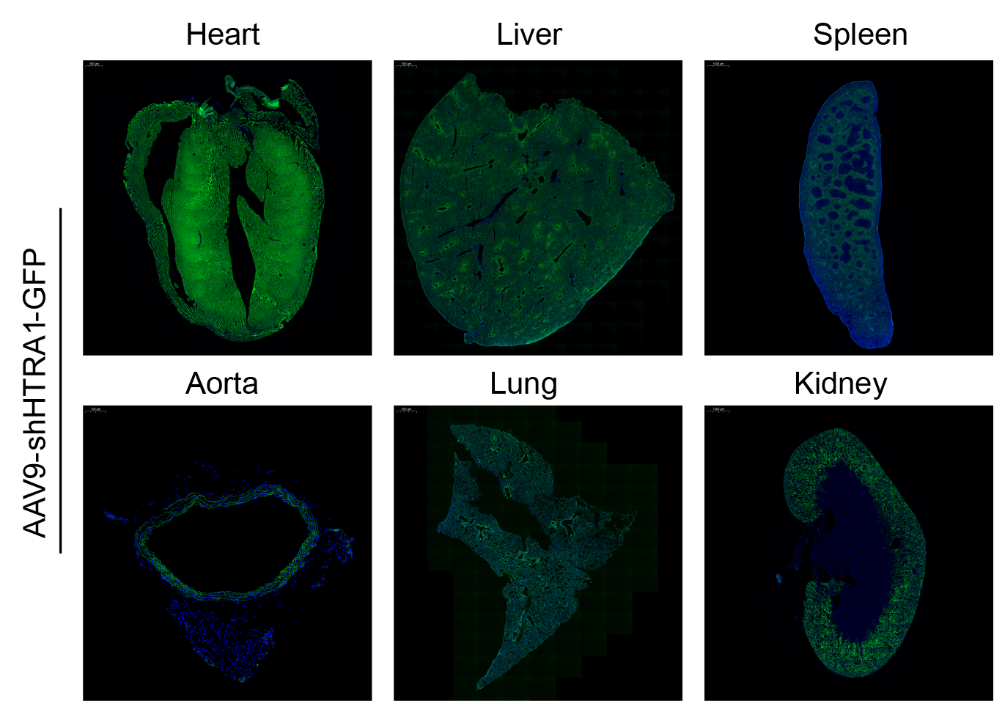
Fig. S3 Frozen section staining of heart, liver, spleen, lung, kidney and aorta after injecting AAV9-shHTRA1-GFP.


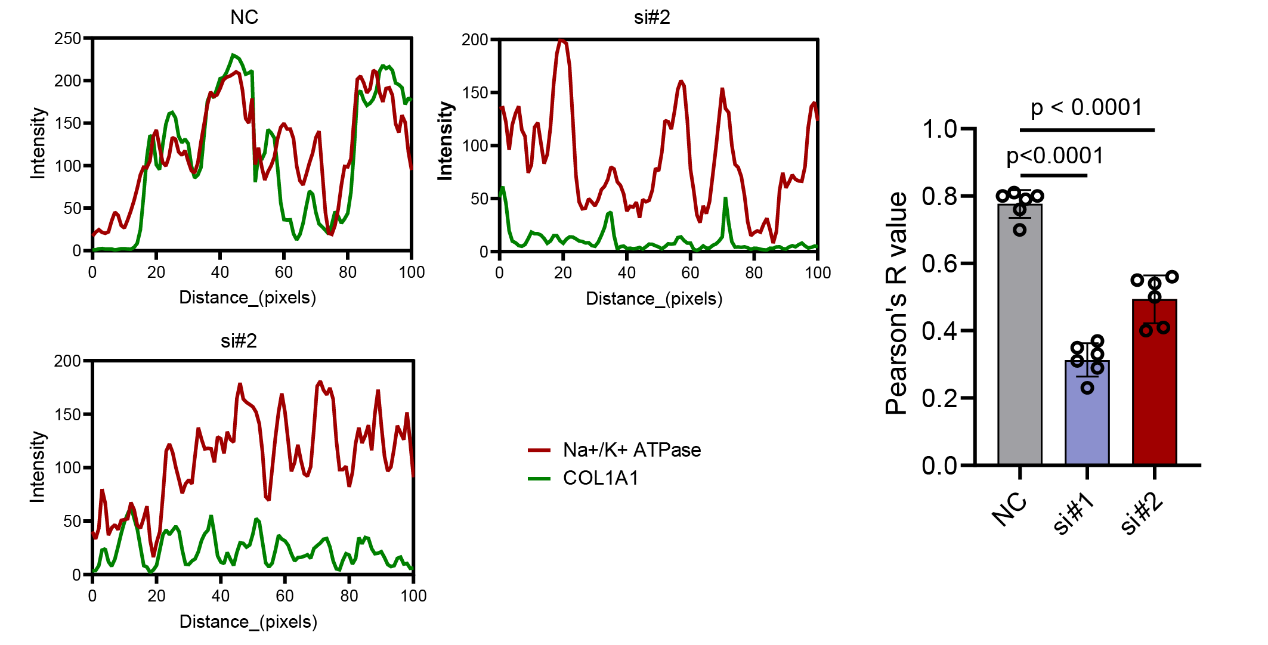


Fig. S4 Quantitative analysis of colocalization of Col1 and Na+/K+ ATP fluorescence.


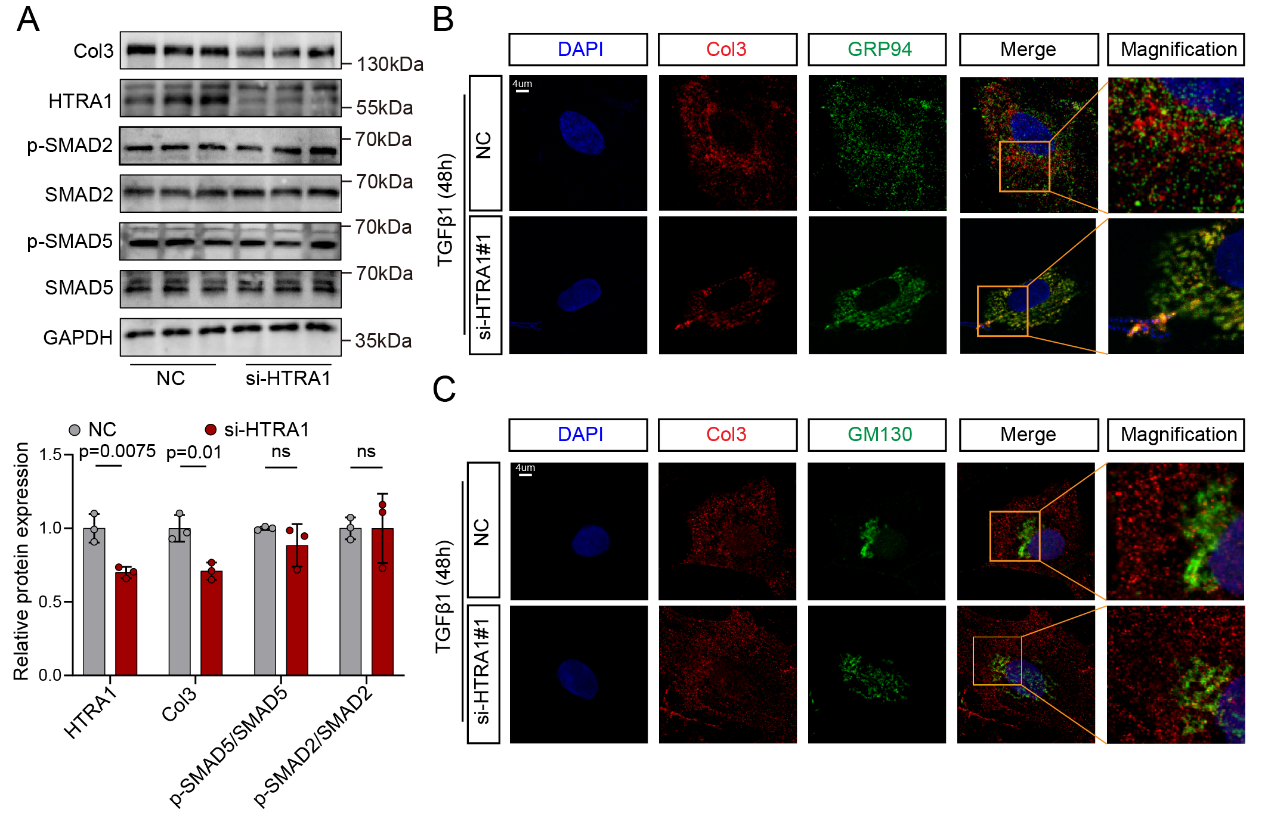


Fig. S5 (A). Representative western blot showing the change of Col3 and p-SMAD2/5 protein expression after HTRA1 inibition. Immunofluorescence assay exhibiting the colocalization of col1 and GRP94 (B) or GM130 (C) in cardiac fibroblasts treated with or without HTRA1 siRNA. Orange box represented a typical colocalization field of view. The scale bar indicates 4um. Middle images showing the intensity of col1 and GRP94/GM130 along with the whit lines.

**
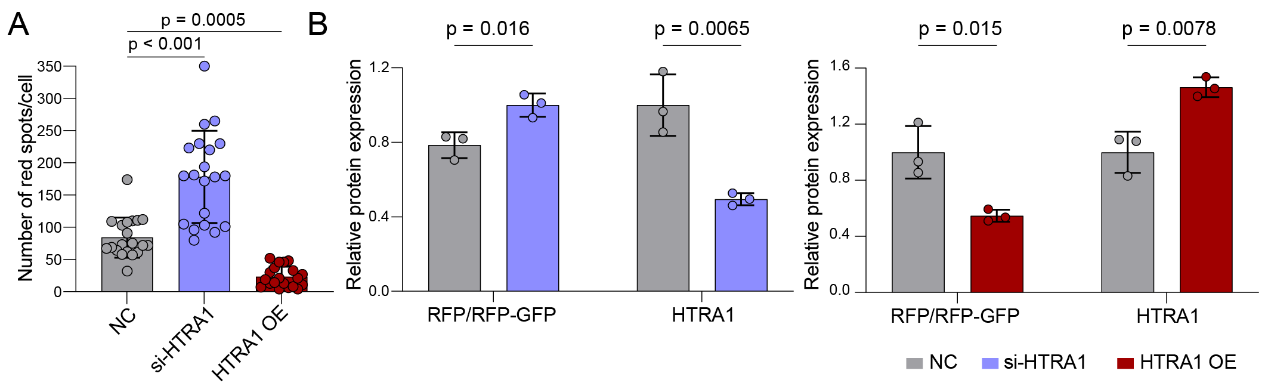
**

**Fig. S6. Quantitative data plots.**

(A). Quantitative data of red spots in different live-cardiac fibroblasts images. (B). Quantitative data showing the difference of RFP/RFP-GFP between NC and si-HTRA1 or HTRA1 plasmid groups.


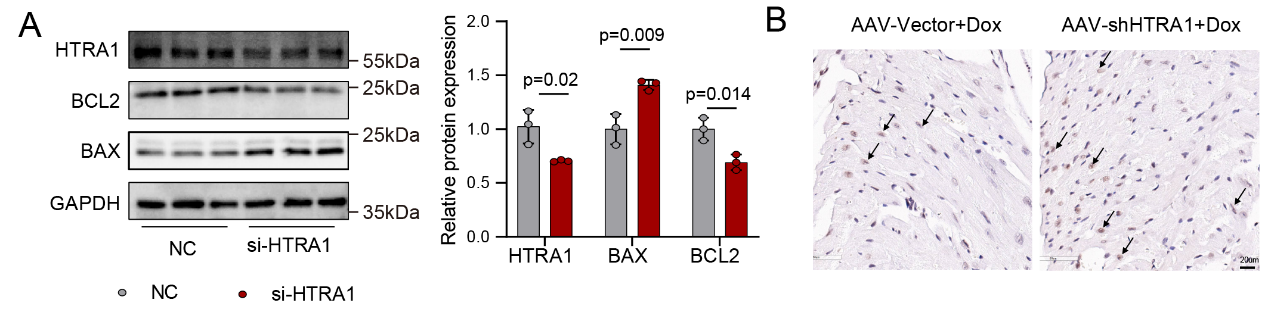


Fig. S7 Representative western blot showing the change of BCL2 and BAX protein expression after HTRA1 inibition. (B). TUNEL staining of mouse heart tissue. The scale bar indicates 20um.

**Table S1. Data sets used in this study.**

| **Data sets** | **Organism** | **PMID** | **Sample size** | **Platform/Technology** |
| --- | --- | --- | --- | --- |
| GSE116250 | Homo sapiens | 30419824 | DCM:NF=37:14 | GPL16791/Illumina HiSeq 2500 |
| GSE57338 | Homo sapiens | 25528681 | DCM:NF=82:136 | GPL11532/Affymetrix Human Gene 1.1 ST Array |
| GSE79962 | Homo sapiens | 28067713 | DCM:NF=9:11 | GPL6244/Affymetrix Human Gene 1.0 ST Array |
| GSE99321 | Homo sapiens | 28724804 | DCM:NF=7:7 | GPL16791/Illumina HiSeq 2500 |
| GSE120895 | Homo sapiens | 31274368 | DCM:NF=47:8 | GPL570 /Affymetrix Human Genome U133 Plus 2.0 Array |
| GSE3585 | Homo sapiens | 17045896 | DCM:NF=7:5 | GPL96/Affymetrix Human Genome U133A Array |
| GSE97642 | Mus musculus | 29230965 | Dox:ctrl=5:5 | GPL6887/Illumina MouseWG-6 v2.0 expression beadchip |

**Table S2. qPCR primer sequences.**

| Gene | Sequence (5'to3') | Organism |
| --- | --- | --- |
| HtrA1 FP | CAAAGCCAAAGAGCTGAAGG | Homo sapiens |
| HtrA1 Rp | ACCATGTTCAGGGTGCTTTC | Homo sapiens |
| GAPDH Fp | CCACCCATGGCAAATTCC | Homo sapiens |
| GAPDH Rp | TCGCTCCTGGAAGATGGTG | Homo sapiens |
| HtrA1 FP | TTATCGCTGATGTGGTGGAG | Rat |
| HtrA1 Rp | AATGAATCCTGACCCACTCG | Rat |
| GAPDH Fp | AGACAGCCGCATCTTCTTGT | Rat |
| GAPDH Rp | CTTGCCGTGGGTAGAGTCAT | Rat |

**Table S3. Antibody information.**

| Antibody | Dilution | Application | Company |
| --- | --- | --- | --- |
| GAPDH (10494-1-AP) | 1:10000 | WB | Proteintech |
| CTGF (25474-1-AP) | 1:1000 | WB | Proteintech |
| α-SMA (19245) | 1:1000 | WB | Cell Signaling Technology |
| α-SMA (19245) | 1:100 | IF | Cell Signaling Technology |
| HTRA1 (55011-1-AP) | 1:1000 | WB | Proteintech |
| HTRA1 (55011-1-AP) | 1:100 | IF | Proteintech |
| Col1 (72026S) | 1:1000 | WB | Cell Signaling Technology |
| Col1 (72026S) | 1:100 | IF | Cell Signaling Technology |
| Fib (15613-1-AP) | 1:1000 | WB | Proteintech |
| GM130 (11308-1-AP) | 1:1000 | WB | Proteintech |
| GM130 (11308-1-AP) | 1:100 | IF | Proteintech |
| GRP94 (14700-1-AP) | 1:1000 | WB | Proteintech |
| GRP94 (14700-1-AP) | 1:100 | IF | Proteintech |
| SEC16A (20025-1-AP) | 1:1000 | WB | Proteintech |
| SEC16A (20025-1-AP) | 1:100 | IF | Proteintech |
| SEC31 (17913-1-AP) | 1:100 | IF | Proteintech |
| Na+/K+ ATP (14418-1-AP) | 1:100 | IF | Proteintech |
| LC3B (2775S) | 1:1000 | WB | Cell Signaling Technology |
| Beclin1 (D40C5) | 1:1000 | WB | Cell Signaling Technology |
| RFP (AE020) | 1:1000 | WB | Abclonal |
| GFP (AE012) | 1:1000 | WB | Abclonal |
| ATF6 (24169-1-AP) | 1:1000 | WB | Proteintech |
| GRP78 (11587-1-AP) | 1:1000 | WB | Proteintech |
| EIF2A (11170-1-AP) | 1:1000 | WB | Proteintech |
| p-EIF2A (28740-1-AP) | 1:1000 | WB | Proteintech |
| IRE1 (27528-1-AP) | 1:1000 | WB | Proteintech |
| p-IRE1 (AP0878) | 1:1000 | WB | Abclonal |
